# Exploring the Reversible Equilibrium State between ^3^CS and ^3^CSS in a Ru(phen)–Naphthalene Diimide Dyad

**DOI:** 10.1021/acs.inorgchem.4c05443

**Published:** 2025-04-21

**Authors:** Lorena Maria Borges Pereira, Diego França de Oliveira, Marco Antonio Tiburcio, Gabriel H. Ribeiro, Carlos André Ferreira Moraes, Flávio Olimpio Sanches Neto, Ademir João Camargo, Leonardo De Boni, Otaciro Rangel Nascimento, Manoel G. P. Homem, Rose Maria Carlos

**Affiliations:** † Departamento de Química, Universidade Federal de São Carlos, CP 676, CEP 13565-905 São Carlos-SP, Brazil; ‡ Instituto de Física de São Carlos, Universidade Estadual de São Paulo, CP 369, CEP 13560-970 São Carlos-SP, Brazil; § Grupo de Química Teórica e Estrutural de Anápolis, Centro de Pesquisa e Pós-Graduação, Universidade Estadual de Goiás, CP 459, CEP 75132-40 Anápolis-GO, Brazil; ∥ Instituto Federal de Educação, Ciência e Tecnologia de Goiás, CEP 72876-601 Goiânia-GO, Brazil

## Abstract

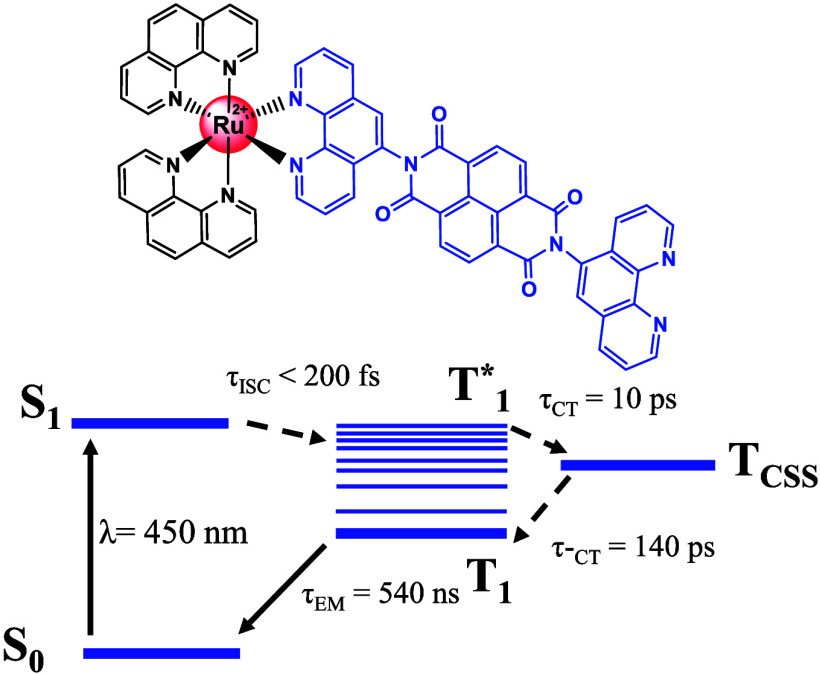

This study explores the dynamics of charge separation (CS) and recombination in the photoinduced electron transfer of the [Ru(phen)_2_(pNDIp)]^2+^ dyad, focusing on the thermal equilibrium between rapid charge separation (CS) and the slower charge-separated state (CSS). The pNDIp component is a naphthalene diimide linked to one of the phen ligands, providing nearly unrestricted orthogonal freedom between the {[Ru(phen)_3_]^2+^} and {pNDIp} units. The investigation employs steady-state and time-resolved spectroscopic techniques, electrochemical methods, and DFT/TD-DFT computational calculations. The results show that selective excitation of the {[Ru(phen)_3_]^2+^} at 450 nm partially quenches the ^3^MLCT emission due to thermal equilibrium with the ^3^CSS state, ^3^{Ru^3+^(phen^•^–^^)_2_(pNDIp)} ⇌ ^3^{Ru^3+^(phen)_2_(pNDIp^•^–^^)}. This equilibrium is attributed to a combination of nonradiative forward (τ_CT_ = 10 ps) and reverse (τ_–CT_ = 140 ps) time decays, driven by the intramolecular charge transfer. The long-lived ^3^MLCT state, the reduced distance between the donor and acceptor, and the vibrational structure of the dyad provide sufficient time for ^3^CS⇌^3^CSS equilibrium. These findings support Marcus theory and highlight key parameters such as −Δ*G*_CS_ = 0.279 eV, λ = 0.49 eV, and H_DA_ = 0.28 eV. Additionally, the dyad’s ability to generate singlet oxygen under 450 nm light suggests potential applications in photodynamic therapy and oxidative processes. Its ability to form radical anion RupNDIp^•^–^^ upon 350 nm light exposure further demonstrates its versatility in photocatalytic applications.

## Abstract 2

The dynamics of charge separation (CS) and recombination in the photoinduced electron transfer of the [(Ru(phen)_2_(pNDIp)]^2+^ dyad, focusing on the thermal equilibrium between rapid charge separation (CS) and the slower charge-separated state (CSS).

## Introduction

There has been considerable interest in photoinduced electron transfer processes of Donor–Acceptor (D–A) dyads for applications in light energy conversion.^1^ The efficiency of these processes depends not only on the components’ responses to the incident light but also on the subsequent conversion of light absorbed into a storable charge-separated excited state (D^•+^–A^•-^). The charge-separated state (CSS) can accumulate sufficient energy for practical organic synthesis and fuel applications.^2−4^ Therefore, a key focus of research has been on controlling the excited-state energy to promote efficient charge separation while minimizing charge recombination, which is crucial for optimizing light energy conversion processes.

Several D–A dyads based on organic chromophores have been developed, including functionalized electron donor/fullerene derivatives, porphyrin derivatives, perylenediimide derivatives, and naphtalenediimides derivatives.^5−11^

A set of four dyads, with triarylamine as the donor and naphthalene diimide as the acceptor, bridged by a meta-conjugated diethynylbenzene bridge with OMe, Me, Cl, and CN substituents, reported by Schäfer et al., reveals photophysics that depend on the bridge substituent. The authors demonstrate that the acceptor substituents increase both charge separation and recombination rates, emphasizing the importance of substituents in tuning charge transfer processes.^12^ The C_3_-symmetric disc-shaped chromophore BTT(NDI)_3_ features electron-accepting naphthalene diimides linked to the electron donor BTT, exhibiting rich photophysics that is dependent on the excitation wavelength. Upon excitation of the BTT moiety, a rapid photoinduced electron transfer occurs from BTT to NDI, resulting in (BTT^•+^-NDI^•–^) with lifetimes up to 3 orders of magnitude longer than (NDI^•+^-NDI^•–^), which forms upon NDI photoexcitation.^13^

As with NDIs, there is growing interest in perylenediimide derivatives.^5−11^ For instance, Chen et al. prepared an orthogonal dyad based on perylenediimide as an electron donor and 4-aminonaphtalimide as an electron acceptor, (PDI-Ny). Excitation on the PDI moiety led to a fast spin–orbit-coupled charge transfer-induced intersystem crossing (SOCT-ISC) with a charge-separated state (τ = 1.7 ps) and charge- recombination (τ = 6.9 ps) in CH_2_Cl_2_: subsequently, deactivation to the ^3^PDI (τ = 175 μs) becomes favorable. The authors conclude that orthogonal geometry is insufficient for achieving efficient SOCT-ISC in compact electron donor/acceptor dyads.^14^ Another strategy, proposed by Chong Wang et al., involved the aggregation of a fullerene-indacenodithiophene dyad. The authors demonstrated that the formation of aggregates leads to a fast-lived charge separation (τ = 0.4 ps) and long-lived CSS (40 μs) facilitated by the presence of an intermediated hot charge transfer state that deactivates into an intramolecular triplet CSS mediated by spin-uncorrelated free carriers.^15^

Our group,^16−18^ along with others,^19,20^ has focused on developing hybrid systems that incorporating Ru(II) metal complexes as electron donors and PDI as electron acceptors. One key advantage of Ru(II) polypyridine complexes is their photophysical properties, which are governed by the lowest energy, long-lived triplet ^3^MLCT state.^21^ Thus, if the CSS originates from the ^3^MLCT state, it retains a triplet spin configuration, which can prolong its lifetime. Additionally, both Ru(II)-polypyridine complexes and PDI exhibit high molar absorptivity, dark and photochemical stability, and the ability to store energy.

In this regard, we reported the photophysical properties of the dyad [Ru(phen)_2_(pPDIp)]^2+^(RupPDIp, phen = 1,10-phenanthroline and pPDIp = perylene pendant group functionalized with one of the coordinated phenanthroline) in CH_3_CN solution. Our strategy was to use the rigid [Ru(phen)_3_]^2+^ structure and planar orientation of pPDIp to promote orthogonal alignment, enhancing charge mobility. The absorption and emission features of both components are preserved in the dyad. Regardless of the excitation wavelength (450 nm for {[Ru(phen)_3_]}^2+^ or 535 nm for pPDIp), the ^3^MLCT state of {[Ru(phen)_3_]}^2+^ is populated. This state then undergoes an intramolecular charge transfer to a short-lived charge-separated state, {Ru^3+^(phen)_2_(pPDIp^•^–^^)}, which decays to the ^3^pPDIp state with a lifetime of 1.8 μs. The dyad efficiently generates singlet oxygen (ϕ = 0.58).^16^

Using the electron-donating properties of pyrrolidine groups (Py), we have designed a dyad that incorporating two pyrrolidine groups in the bay positions of pPDIp.^18^ This modification shifts the absorption and emission of the new dyad, RuPDI-Py, to the red region (with absorption at 720 nm and emission at 780 nm) while maintaining a donor–acceptor distance similar to that of RuPDIp.

The photophysical properties of RuPDI-Py are dominated by the fluorescence of the PDI-Py component. Quenching of the ^3^MLCT state provides a pathway to the formation of a long-lived charge-separated state, {Ru^3+^(phen)_2_(PDI-Py^•-^)}, which has a lifetime of 4 μs. This charge-separated state then converts to the long-lived ^3^PDI–PY state with a lifetime of 28 μs, which photosensitizes singlet oxygen formation.^18^

Despite promising results, achieving a stable charge-separated state remains challenging due to the faster charge recombination rate from the ^3^CSS state to the ^3^PDI state.

**Scheme 1:**
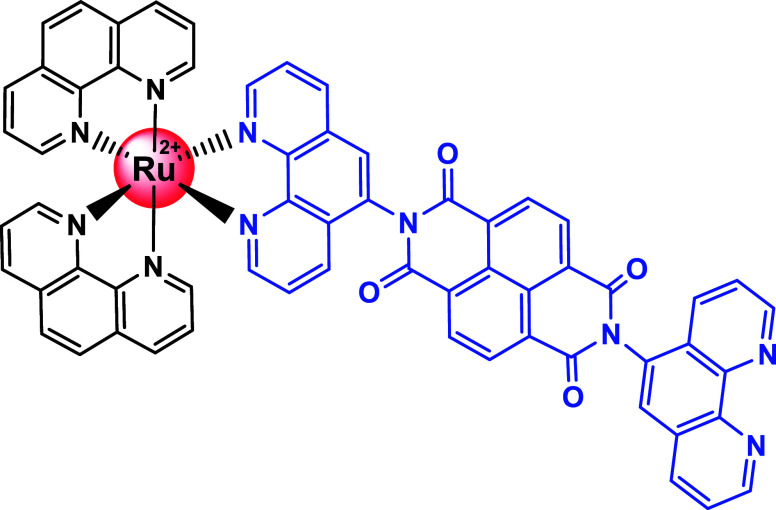
Molecular Structure of the RupNDIp Dyad

This work addresses this issue by replacing PDI with a naphthalene-diimide (NDI) group (Scheme S1), resulting in the dyad [Ru(phen)_2_(pNDIp)]^2+^(RupNDIp, phen = 1,10-phenanthroline and pNDIp = naphthalene pendant group functionalized with one of the coordinated phenanthroline), Scheme 1. We hypothesize that reducing the distance between the donor and acceptor in the RupNDIp dyad will bring the ^3^CS and ^3^CSS states closer in energy, thereby stabilizing the charge-separated state by offering sufficient time for equilibrium between them.

The RupNDIp dyad is easy to prepare in high yield (65%), soluble in DMSO and acetonitrile solution, and stable in the solid state and solution in the dark and the light. The photophysical studies were investigated in DMSO solution using steady-state and time-resolved spectroscopic and electrochemical techniques, as well as DFT and TD-DFT calculations.

Our results show a significant effect of structural modifications compared to similar dyads, RuPDI and RuPDI-Py. By shortening the distance between the donor and acceptor components, it was possible to tune the excited-state dynamics to achieve thermal equilibrium between the long-lived ^3^MLCT and the ^3^CSS states.

This suggests that optimizing spatial arrangement and electronic properties can enhance charge transfer efficiency and stability in the charge-separated state. Such modifications can be crucial for fine-tuning the photocatalytic properties.

In this context, the dyad’s ability to generate singlet oxygen under 450 nm light suggests potential in photodynamic therapy and oxidative processes. Additionally, the formation of the radical anion RupNDIp^•^–^^ under 350 nm light enables participation of the dyad in the electron transfer process. This dual capability enhances the dyad’s versatility in photocatalytic applications.

## Experimental Section

### General Methods and Materials

All reagents used in the synthesis were sourced from Sigma-Aldrich, and the solvents used in the spectroscopic analyses were of HPLC grade. The synthetic procedures and characterization of the synthesized compounds are described in the Supporting Information.

### Photophysical Studies

UV–vis absorption spectra were recorded with an Agilent Technologies 8543 spectrophotometer using quartz cells with an optical path length of 1.0 cm. The emission and excitation spectra were recorded by using a SHIMADZU RF-5301PC spectrometer. The emission spectrum was not corrected for photomultiplier responses. The emission quantum yield of the RupNDIp dyad was measured using [Ru(bpy)_3_]Cl_2_ in N_2_-saturated (Φ_EM_ = 0.042 ± 0.002) and in air-equilibrated (Φ_EM_ = 0.028 ± 0.002) in water as a reference.^22^

### Transient Absorption Spectroscopy

Transient absorption measurements were performed in a pump and probe experimental setup, using a Yb:KGW femtosecond pulsed laser (Light conversion, Model PHAROS) with a fundamental emission at 1030 nm. Pump pulses were generated using an optical parametric oscillator that allows for wavelength tuning between 220 nm and 3 μm. The pump passes through a temporal delay line that can induce a delay of up to 600 ps. A chopper is synchronized with the laser and either blocks the pump pulse (pump-OFF) or allows it to pass (pump-ON). If the pump crosses the chopper, then it will hit the sample and create an excited-state population. Probe pulses are made using white light continuum generation in a Saphire crystal. Probe pulse hits the sample at the same place as the pump, and the transmitted light is measured in a spectrometer. Comparing the transmitted probe spectrum during pump-ON and pump-OFF measurements, it is possible to observe the transient excited-state dynamics of the sample as a function of the time between the pump and probe. All of the samples were measured at the same absorbance (0.7) at the pump wavelength. A more complete description of the experimental setup is presented in the Supporting Information.

### Electrochemical Measurements

Cyclic voltammetry measurements were conducted at room temperature in dimethyl sulfoxide (DMSO) and acetonitrile (CH_3_CN), purged with N_2_, using an EcoChemie Autolab 30 potentiostat in a three-electrode setup. The working electrode consisted of a glassy carbon electrode (3 mm diameter), the counter electrode was a platinum wire electrode, and a silver wire was used as a quasi-reference electrode. All electrodes were polished on a felt pad with a 0.05 or 0.3 μm alumina suspension and sonicated in deionized water for 1 min before each experiment. Tetrabutylammonium hexafluorophosphate (TBAPF_6_, 0.1 M) was added to the solution as a supporting electrolyte.

UV–vis spectroelectrochemical experiments were performed in an electrochemical cell equipped with quartz windows, two platinum minigrids as working and counter electrodes, and a silver wire as a quasi-reference electrode. The solutions used in the spectroelectrochemical experiments were prepared as described previously for cyclic voltammetry. UV–vis–NIR absorption spectra were recorded upon application of a given potential using an Agilent Technologies 8543 diode array spectrophotometer.

### Singlet Oxygen Measurements

Singlet oxygen masurements by UV–vis absorption spectroscopy were performed using a 3 mL solution containing 100 μmol·L^–1^ of 9,10-anthracenediyl-bis(methylene) dimalonic acid (ABDA) and 20 μmol·L^–1^ of RupNDIp or [Ru(phen)_3_]^2+^ in DMSO. The initial absorbance (*A*_0_) at 380 nm was recorded using 100 μmol·L^–1^ of ABDA in DMSO as the reference solution. The mixture was then irradiated with 450 nm light, and the absorbance (*A*_t_) at 380 nm was measured at predetermined time intervals.

The singlet oxygen emission spectra in the NIR region were acquired using an FLSP 920 photon counter (Edinburgh Instruments, Edinburgh, UK) consisting of a H10330A-45 NIR-PMT detector (Hamamatsu, Japan) coupled to a thermoelectric cooling module maintained at −60 °C to reduce dark current. The singlet oxygen emission was processed through a monochromator (TMS/DTMS300, Edinburgh Analytical Instruments, UK) equipped with a diffraction grating capable of selecting wavelengths in the infrared region. The output from the photomultiplier tube was connected to a computer, and the signal was acquired. The monochromator was controlled, and data were acquired using F-900 software, version 6.22 (Edinburgh Analytical Instruments, Livingston, UK). The NIR emission lifetime at 1.270 nm was measured by using a single photon counting system with a band-pass filter placed between the cuvette and the photomultiplier. All sample components were mixed and transferred to a glass cuvette (35 × 7 × 55 mm^3^) maintained at 25 °C.

### Electron Paramagnetic Resonance (EPR) Spectroscopy

EPR measurements were acquired using a Varian E109 X-band spectrometer operating at 9.5 GHz. The spectra of pNDIp from the RupNDIp dyad in the dark and after photolysis were obtained using an MgO standard doped with Cr(III). Each solution was irradiated with 350 and 450 nm light, both aerated and deaerated. The EPR conditions were as follows: microwave power, 1 mW; modulation amplitude, 0.1 G; time constant,0.064s, scan time 60 s; 1 scan.

## Results and Discussion

### Synthesis and Characterization

The synthesis of the pNDIp was adapted from the previously reported methods for the structurally substituted NDI compounds,^23^ described in the Support Information (SI). The RupNDIp dyad was synthesized via the reaction between precursor complex *cis-*[Ru(phen)_2_Cl_2_] (0.1 g, 0.18 mmol) and pNDIp (0.17 g, 0.25 mmol) in dimethylformamide (DMF), using ammonium hexafluorophosphate (NH_4_PF_6_) (0.062 g, 0.374 mmol) as the counterion, as detailed in Support Information. The resulting solid was filtered and subsequently purified. For this purpose, it was dissolved in acetone, and the resulting solution was centrifuged to remove the excess free pNDIp ligand, as detected by UV–vis spectroscopy. The solution was then rotary evaporated, and the obtained compound was dried under vacuum, yielding a dark red solid.

The chemical structures of the pNDIp compound and RupNDIp dyad have been confirmed by ESI mass spectroscopy (Figures S1 and S6) and 1D and 2D NMR (Figures S2–S5 and S7–S10).

Figures S1 and S6 display the ESI-MS spectra of the pNDIp compound and the RupNDIp dyad, respectively. The molecular ion peaks for pNDIp appear at *m*/*z* 623.1481 [M + H]^+^ and [M + Na]^+^, while for the RupNDIp dyad, the peak is observed at *m*/*z* 542.0910 [M]^2+^, confirming the synthesized structures.

The combined 1D and 2D-NMR experiments allowed ^1^H and ^13^C signal attribution, and the integration ratios for pNDIp and RupNDIp dyad (Tables S1 and S2) were consistent with the proposed structures.

^1^H NMR spectrum (Figure S2) of the free pNDIp compound shows a pattern of signals consistent with equivalent phenanthroline moieties, with four resolved resonances in the downfield region. The singlet resonance at δ 8.28 is referent to equivalent hydrogens of the naphtalenediimide moiety from pNDIp core, Table S1. In relation to the RupNDIp dyad, the pattern of resonances in the ^1^H and ^13^C NMR spectra is consistent with a low symmetry of the dyad, as shown in Figures S7–S10. The ^1^H NMR spectrum displays different patterns of resonances in the downfield region for the hydrogens of the dyad, which is marked by overlaps of respective signals of the rings from diimines ligands coordinated to the metal. Except for H8ʼ and H6ʼ, it is important to highlight that each signal of the phen moiety is remarkably shifted upfield (Δδ: −0.17 to −0.57) compared to the corresponding hydrogens on the noncoordinated phen. Upon the coordination of the pNDIp to the Ru(II) center, the chemical shifts of the resonances of the phen moiety from pNDIp bound to Ru(II) center were similar to that of the phen ligand bound to the metal center. On the other hand, the chemical shifts of the hydrogens noncoordinated phen moiety from pNDIp were not affected by the coordination to metal, and the resonances were very similar to free pNDIp compound.

### UV–vis Absorption, Emission, and Electrochemical Studies

**Figure 1:**
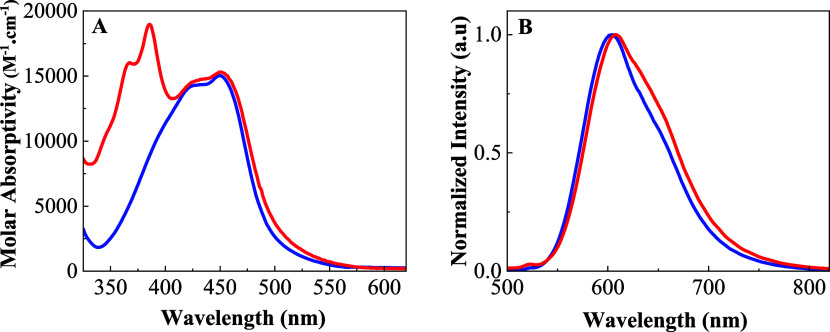
Visible region spectrum. (A) Absorption and (B) emission (λ_EX_ = 450 nm) spectrum of [Ru(phen)_3_]^2+^ (blue) and RupNDIp dyad (red) in DMSO solution (*c* = 20 μmol L^–1^).

The UV–vis absorption spectra of [Ru(phen)_3_]^2+^ and RupNDIp in DMSO solution are shown in Figure 1A, and the data are summarized in Table S4. The [Ru(phen)_3_]^2+^ complex shows strong UV absorption, mostly π–π* in origin (Figure S14A), and a broad absorption in the visible region around 350–550 nm attributed to metal-to-ligand charge transfer (^1^MLCT) electronic transitions similar to the reported spectrum.^24^ The pNDIp compound exhibits a vibrational resolved spectrum in the UV–vis region (300–400 nm) typical for compounds of this type,^25,26^Figure S14B. The absorption spectrum of RupNDIp is almost the sum of the spectra for the {[Ru(phen)_3_]^2+^} and {pNDIp} components, indicating weak interactions between them. This is attributed to the orthogonal geometry between the components, as demonstrated by DFT calculation (see details in the SI). The lowest energy absorption band of the RupNDIp is assigned to the ^1^MLCT electronic transitions of the [Ru(phen)_3_]^2+^ therefore selective excitation of the Ru(II) moiety is expected in this wavelength region. In the high energy absorption (<400 nm), both components absorb the incident light with a repartition of 78% of the excitation energy being absorbed by the pNDIp component. These results agree with the results from the TD-DFT calculations, as detailed in the SI.

The emission spectrum of RupNDIp is characterized by a broad emission band with a maximum at 610 nm that resembles the ^3^MLCT phosphorescence band of the [Ru(phen)_3_]^2+^ complex, Figure 1B. The emission spectrum is independent of the excitation wavelength, Figure S15A. However, the emission intensity depends on the presence of oxygen, with a nearly 75% decrease in intensity observed in the air-equilibrated DMSO solution compared to the deaerated solution (Figure S15B). The emission spectrum is also relatively dependent on the solvent, with a shift of 20 nm from CH_2_Cl_2_ (586 nm) to DMSO (610 nm), Figure S16. Compared to the [Ru(phen)_3_]^2+^ complex (Φ_EM_ = 0.05, τ_em_ = 950 ns), the emission lifetime of RupNDIp (Φ_EM_ = 0.0075, τ_EM_ = 540 ns) in a deaerated DMSO solution is approximately 55% shorter, and its emission quantum yield is reduced by 75% (Table S4). These results contrast with those previously reported by our group for the emission spectra of RupPDIp (^3^MLCT and ^1^pPDIp) and RuPDI-Py (^1^pPDIp).

Figures S17 and S18 show the cyclic voltammograms of pNDIp in DMSO and the RupNDIp dyad in DMSO and acetonitrile. In DMSO (Figure S18A), pNDIp is easily reduced to pNDIp^•–^, with no significant change in redox potential upon coordination to Ru(II), indicating the electron-withdrawing nature of NDI does not affect the dyad’s redox potential.

**Figure 2:**
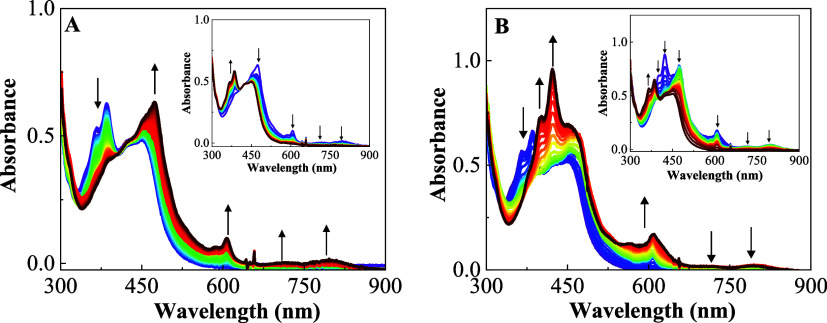
Spectroelectrochemical analysis for RupNDIp dyad degassed DMSO (TBAPF_6_, 0.1 M) at applied potentials (A) *E*= −0.37 (V vs Ag^+^/Ag wire as pseudoreference); inset plot of at applied potential 0 V and (B) *E* = −0.73 (V vs Ag^+^/Ag wire as pseudoreference); inset plot of at applied potential 0 V.

The UV–vis spectroelectrochemical experiments of pNDIp (Figure S19) and the RupNDIp dyad in deaerated DMSO are shown in Figure 2. At the first reduction potential (−0.37 V), the RupNDIp dyad forms new peaks at 477, 610, 717, and 796 nm, corresponding to the pNDIp^•–^ radical anion (Figure 2A). At −0.73 V, the RupNDIp^2^ – dianion forms (Figure 2B). Both the radical anion and dianion are stable in deaerated DMSO. As shown in Figure 2 inset plot, the oxidation of the corresponding anion radical and dianion pNDIp moieties in the dyad is reversible. Similar results were observed for pNDIp (Figure S19).

### Picosecond Transient Absorption Spectroscopic Investigations

**Figure 3:**
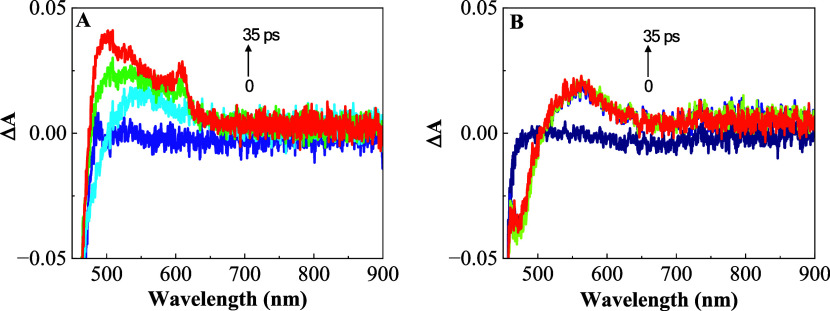
Femtoseconds transient absorption graphs spectra for different time delays in DMSO with λ_EX_ = 450 nm (A) RupNDIp dyad and (B) [Ru(phen)_3_]^2+^ complex.

After exploring the absorption and electrochemical properties of pNDIp and RupNDIp we investigated the transient absorption spectra (TA) of RupNDIp to understand the emission quenching experiments. The emission of RupNDIp was evaluated using ps-transient absorption spectra in DMSO, with a 450 nm excitation pulse of 220 fs duration, Figure 3A. For comparison, the transient absorption of the [Ru(phen)_3_]^2+^ complex was also measured under identical conditions, Figure 3B. The spectral behavior of the [Ru(phen)_3_]^2+^ complex for 1.6 to 35 ps after excitation is characterized by an absorption band at 550 nm. This band appears instantly after excitation and does not seem to decay after 600 ps, indicating that it represents an excited-state absorption (ESA) of the long-lived triplet state of the [Ru(phen)_3_]^2+^ complex, Table S4. The transient absorption spectra of RupNDIp shows the same band at 550 nm now with two additional bands at 497 and 610 nm. The band near 450 nm is distorted due to the mixing of the ESA signal with the ground state bleach negative signal. The [Ru(phen)_3_]^2+^ complex and RupNDIp dyad difference spectrum (Figure S20) taken after 36 ps excitation resulted in a strong absorption at 484 and 600 nm which indicates the formation of the radical anion RupNDIp^•-^.^27^ Moreover, the transient absorption spectrum is in good agreement with the UV–vis spectroelectrochemical measurement of the first reduction (Figures 2A and S21) and photolysis at 350 nm of the RupNDIp dyad (described below). Between 36 and 600 ps it is possible observe the decay of the bands at 497 and 610 nm, indicating the disappearing of the anion (Figure S22). The band at 550 nm remains unchanged during 600 ps, as expected for a long-lived triplet state. The ESA spectrum profile was independent of the solvent (DMSO, acetonitrile), and graphs in acetonitrile can be seen in Figures S23 and S24. One may notice that the RupNDIp presented higher Δ*A* for the bands in acetonitrile then in DMSO, but the comparison of Δ*A* between different transient absorption measurements is not necessarily related of the material properties. Small changes in the pump or probe beams could have a big influence on these amplitudes since the setup is not normalized for their intensity.

The temporal behavior of the 484 and 600 nm transient absorption in DMSO could be fit by two exponentials, one negative and one positive (Figure S25). The negative one (charging behavior) (τ_CT_∼10 ± 1 ps) represents the formation of the ESA by separation of charge, creating the ^3^CSS. The positive one (decaying behavior) (τ_–CT_∼140 ± 10 ps) represents the ESA’s decay by the charge recombination, diminishing the ^3^CSS. For RupNDIp dissolved in acetonitrile the charging and decaying lifetimes were τ_CT_∼14 ± 1 ps and τ_–CT_∼110 ± 10 ps, respectively. Considering the different times, charge formation is slightly more favorable in DMSO.

**Scheme 2:**
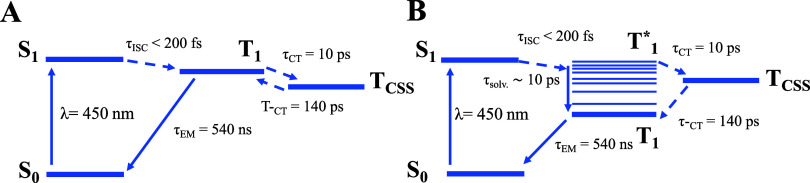
Energy Level Diagram Containing the Possible Transitions and Their Respective Decay Paths with Explicit Lifetimes for (A) a Model That Doesn’t Consider Vibrational Relaxation and (B) a Model That Considers the Vibrational Relaxation of the Locally Excited Triplet State

As the charge formation lifetime (τ_CT_) and the charge recombination lifetime (τ_–CT_) are different, both processes may not involve the same energy levels, as has been observed in other works.^28^ This indicates that a vibrational cooling decay path is crucial to understanding the population dynamics in the course. However, as this energy decay process does not change the electronic state of the molecule, its effect on the transient absorption is minimal. It can hardly be observed in the case of these transient species. To understand how vibrational cooling affects population dynamics, a simulation was made in PYTHON3 using two different energy level models presented in Scheme 2. The simulation runs interactions of the rate equations for the variation of each of the state’s populations at intervals of 100 fs for up to 1 μs. The rate equations that describe the populations’ dynamics are different for each of the proposed diagrams in Scheme 2.

Scheme 2A considers that no vibrational cooling happens in the locally excited triplet state (*T*_1_). In the scheme, τ_EM_ is the phosphorescence lifetime, τ_CT_ is the charge separation lifetime, and τ_–CS_ is the charge recombination lifetime. The values of τ_EM_, τ_CT_ and τ_–CT_ have already been determined by the studies of the phosphorescence time decay and the transient absorption measurements described above. The rate equations that describe the variation of each of the states population for this model are presented in the group of equations shown in eq 1:
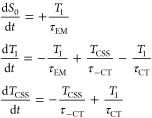
1In the rate equations, *S*_0_, *T*_1_, and *T*_CSS_ are the populations of the ground state, the local excited triplet state, and the charge-separated state, respectively. The initial condition for this simulation is set as *S*_0_ = 0, *T*_1_ = 1, and *T*_CSS_ = 0, which represents the instant after full intersystem crossing (<ps after excitation).

Scheme 2B represents a system in which the locally excited triplet state cools before returning to the ground state. τ_solv._ is the solvation and vibrational cooling lifetime and its value is expected to be in the order of a few picoseconds and may vary depending on solvent and temperature.^29^ We used a value of 10 ps as it gives values that accurately reproduce the observed effects. The rate equations that describe the variation of each of the states’ populations for this model are presented in group of equations shown in eq 2:
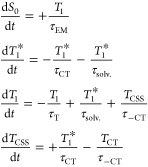
2In the rate equations, *T*_1_^*^ is the locally excited triplet state with additional vibrational energy, while the *T*_1_ state is the locally excited triplet state after full vibrational cooling. The initial condition for this simulation is set as *S*_0_ = 0, *T*_1_* = 1, *T*_1_ = 0, and *T*_CSS_ = 0, which represents the instant after full intersystem crossing (<ps after excitation).

**Figure 4:**
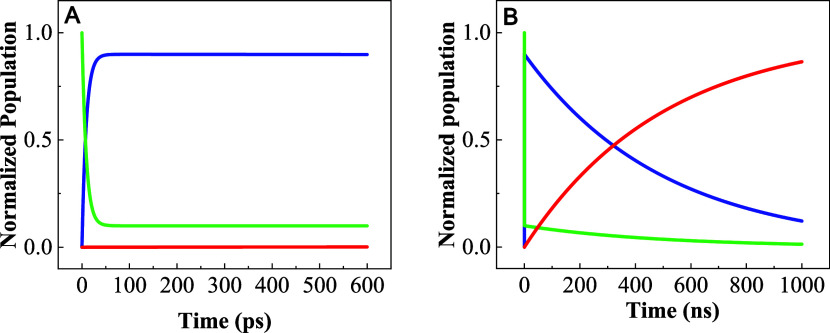
Relative population of Ground State (*S*_0_, red), locally excited triplet state (*T*_1_, green), and charge separation state (CSS, blue) as a function of time up to (A) 600 ps and (B) 1000 ps.

The results of each of the simulated models are presented as a graph of the population over time. Figure 4 shows the model’s population dynamics from 0 to 600 ps excluding vibrational cooling effects, while Figure 4B shows the dynamics from 0 to 1 μs. It can be seen that this model does not resemble the behavior observed experimentally. The transient population of the charge separation state creates an equilibrium with the triplet state that lasts for over 1 μs. If this were to be true, then both the ESA of the CSS and the phosphorescence emission should decay within the same time, which is not the case.

**Figure 5:**
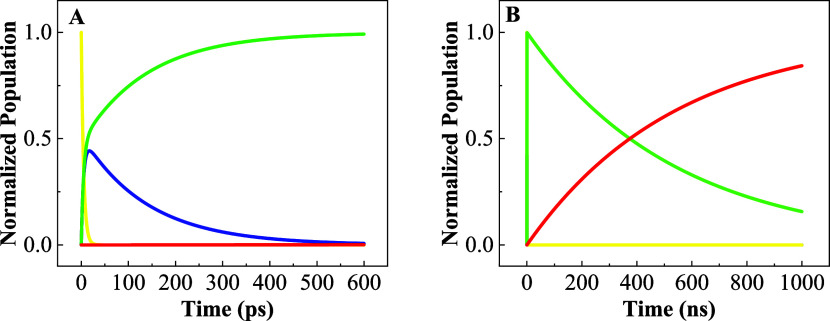
Relative population of ground state (*S*_0_, red), locally excited triplet state with vibrational energy (*T*_1_*, yellow), locally excited triplet state without vibrational energy (*T*_1_, green), and charge separation state (CSS, blue) as a function of time up to (A) 600 ps and (B) 1 μs.

Figure 5 shows the population dynamic from 0 to 600 ps for the model that includes vibrational cooling effects, while Figure 5B shows the dynamics from 0 to 1 μs for this same model. This model resembles much better the behavior observed experimentally. First, the CSS population has an increase and decrease within the first nanosecond of the dynamics. After that, the triplet state persists for over 1 μs, as it is observed in the phosphorescence emission decay.

The results show that the rapid decay time of the charge separation state and the long-lived phosphorescence decay cannot be simultaneously justified in a system that does not consider the vibrational cooling of the triplet state.

**Scheme 3:**
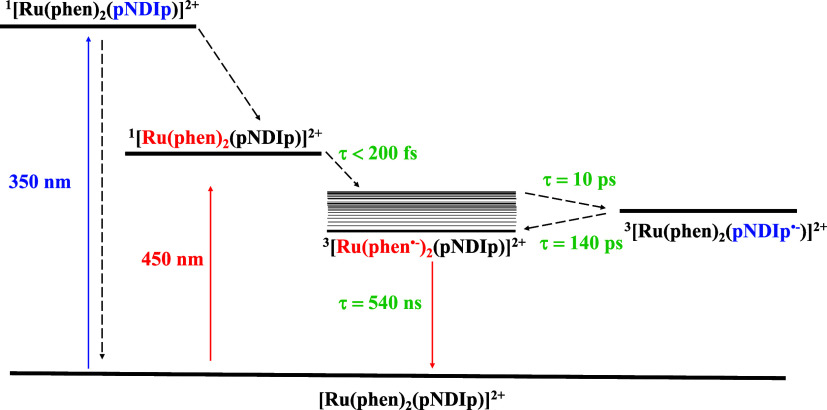
Qualitative Energy–Level Diagram of RupNDIp Dyad in DMSO blue: excitation at 350 nm; red: excitation at 450 nm.

Scheme 3 summarizes the photophysical properties of the RupNDIp. Excitation at 350 nm populates the ^1^pNDIp (3.53 eV) component, which decays to the ground state by an internal conversion process. An alternative decay pathway of ^1^pNDIp is to transfer its excitation energy to the ^1^MLCT (3.53 eV) of the {[Ru(phen)_3_]^2+^} component followed by a subsequent fast intersystem crossing into the ^3^MLCT.

Photoexcitation of RupNDIp at 450 nm where only the [Ru(phen)_3_]^2+^ component absorbs light, results in the population of the ^3^MLCT emissive state. The partial quenching of the ^3^MLCT emission is attributed to two different decay processes: a nonradiative decay process to produce singlet oxygen (described below) and a fast intramolecular electron transfer to the triplet charge separate state {Ru^3+^(phen)_2_(pNDIp^•-^)} accompanied by forward (τ_CT_ = 10 ps) and reverse nonradiative processes (τ_–CT_ = 140 ps).

The dynamic of photoinduced electron transfer of RupNDIp was analyzed by the Marcus Theory of electron transfer.^4^ Specifically, the driving force for photoinduced electron transfer (*Δ*G*_CT_), the reorganization energy (λ), and the electronic coupling (H_DA_) between the [Ru(phen)_3_]^2+^ and pNDIp components were determined as described in the Support Information.

Table 1 summarizes the results obtained. The results indicate a thermodynamic favorable photoinduced charge separation *Δ*G*_CT_ = −0.279 eV and a low reorganization energy of 0.49 eV.^30^ The driving force is smaller than the value of *Δ*G*_CT_ for a Rhodamine-NDI derivative in other studies with smaller D–A distances,^31^ and the reorganization energy is also smaller when compared to theoretical values found for NDI derivatives,^32^ indicating that for RupNDIp electron transport mobility is highly favored. These data indicate that the dyad’s charge separation process (–λ < *Δ*G*_CT_ < 0) is in the normal region of the Marcus parabola. Despite the approximations made, particularly the assumption that the internal contribution (λ_i_) is negligible, the results suggest that the RupNDIp dyad exhibits efficient charge separation under thermodynamically favorable conditions, aligning well with predictions from the Marcus Theory.

**Table 1:** Reduction Potential and Driving Force for the Charge Separation (CS), for RupNDIp, in an Acetonitrile Solution at Room Temperature^a^

*E*_1/2_(Ru(III)/Ru(II)) (V vs SCE)	*E*_1/2_(pNDIp/pNDIp^–^) (V vs SCE)	*W*_p_	*ΔG_CT_	*l*_o_	*H*_DA_
1.035	–0.660	0.038	–0.279	0.49	0.28

a All values are given in eV.

### Photochemical Studies

The photochemical activation of singlet oxygen (^1^O_2_) was evaluated for the RupNDIp dyad with light at 450 nm. For comparison, the experiment was also carried out for the [Ru(phen)_3_]^2+^complex, Figures S26 and S27.

**Figure 6:**
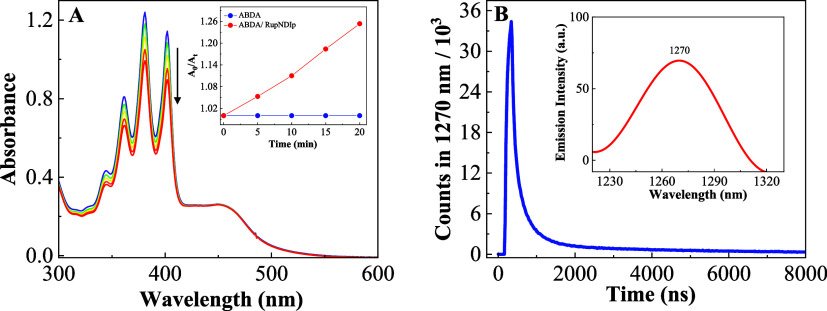
(A) Photolysis of ABDA (100 μM) in the presence of RupNDIp (20 μmol L^–1^) with 450 nm light in DMSO, total irradiation time = 20 min; inset plot of ABDA consumed at 380 nm versus irradiation time. (B) Luminescence intensity decay of singlet oxygen in DMSO at 1270 nm (λ_EX_ = 450 nm); inset emission spectrum of ^1^O_2_ (λ_EX_ = 450 nm) in a DMSO solution of RupNDIp.

Figure 6A illustrates the spectroscopic changes seen when a DMSO solution of RupNDIp was irradiated at 450 nm in the presence of the singlet oxygen probe of 9,10-anthracenyl-bis (methylene)dimalonic acid (ABDA). The spectra show a progressive depletion of the absorption band of ABDA with the irradiation time. The plots of the changes of absorption maximum of ABDA at 380 nm versus the irradiation time, were linear, implying a one-to-one correspondence. This experiment was not carried out for the pNDIp compound at 350 nm because ABDA is photochemically reactive at this wavelength.

The formation of singlet oxygen by RupNDIp (Abs = 0.3; λ_EX_ = 450 nm) in air-equilibrated solution was also evidenced directly by its characteristic phosphorescence spectrum with a maximum of 1270 nm. The singlet oxygen lifetime of 0.370 μs was determined from the curve of phosphorescence decay at 1270 nm (Figure 6B). This value is slightly lower than that determined for [Ru(phen)_3_]^2+^, 0.40 μs, (Figure S27) prepared under the same conditions, which may be due to the competing population of the ^3^CSS. All attempts to directly detect singlet oxygen from the pNDIp compound upon excitation at 350 nm failed, Figure S28.

**Figure 7:**
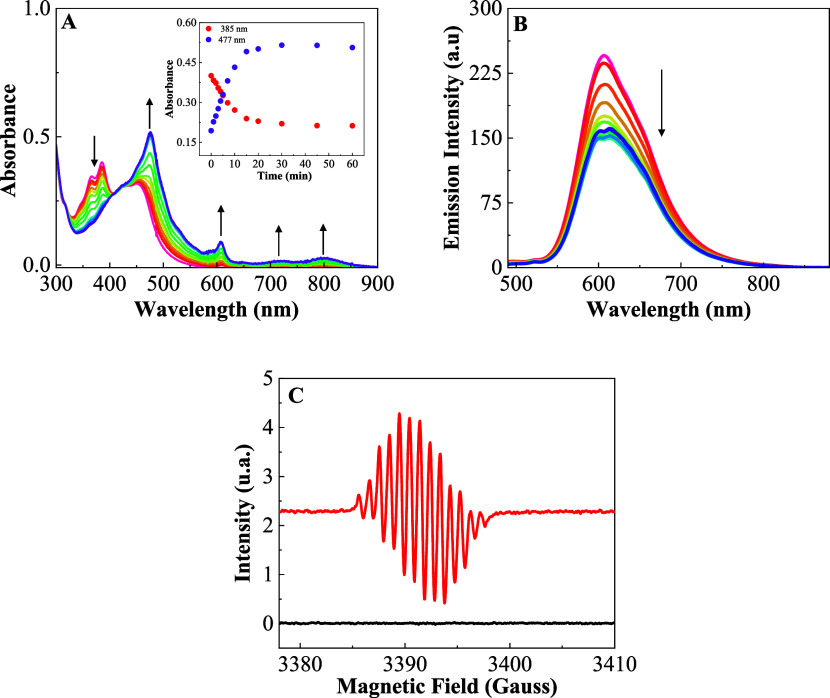
Photolysis of the RupNDIp (20 μmol L^–1^) with 350 nm light (*I*_0_ = 4.09 × 10^–9^ Einstein.s^–1^) in DMSO, total irradiation time = 60 min: (A) Changes in the UV–vis absorption spectra; inset Plot of absorbance at 477 and 385 nm versus irradiation time, (B) changes in the emission spectra (λ_EX_ = 450 nm), and (C) EPR spectrum in the dark (black) and after 60 min time irradiation (red).

The pNDIp compound and RupNDIp dyad are photoreactive at 350 nm in deaerated DMSO solution, and the changes in their UV–vis absorption and emission spectra as well as in the EPR spectra are consistent with the formation of the radical pNDIp^•-^ and RupNDIp^•-^, respectively. Irradiation of the RupNDIp dyad with 350 nm light resulted in the decreased of the pNDIp component absorption and the concomitant appearance of new bands in the visible and near IR region, characteristics of the formation of the [Ru(phen)_2_(pNDIp^•–^)]^2+^ radical anion, Figure 7A. This behavior parallels what was seen with the pNDIp compound, (Figure S29A) highlighting a similar mechanism of electron transfer to anion radical formation upon irradiation.

The absorption of the {[Ru(phen)_3_]}^2+^ component remains constant during irradiation of the RupNDIp, indicating that this component does not participate in the radical formation, allowing for the direct observation of the pNDIp component changes, Figure 7A. The decrease in emission intensity by approximately 50% with irradiation at 350 nm may be related to competition for the 450 nm light between {[Ru(phen)_3_]}^2+^ and RupNDIp^•^–^^, Figure 7B.

The EPR spectrum measured at the end of photolysis shows the formation of a well-resolved 13-line EPR spectrum centered at *g* = 2.0041 attributed to pNDIp^•–^, Figure S29B. The amount of radical anion estimated through integration of the EPR signals was 4 times greater in the pNDIp component than in the RupNDIp dyad. The EPR simulated spectrum calculated by adjusting the parameters with four equivalent hydrogen nuclei and two equivalent nitrogen nuclei confirmed the delocalization of the unpaired electron in the aromatic rings of the pNDIp, Figure S29B. The simulation was obtained using a Gaussian line shape with line width of 0.26 G and hyperfine parameters 1.94 G for the two equivalent nitrogen nuclei and 0.96 G, for the four equivalent hydrogen nuclei.^33^

It should be noted that photolysis at 450 nm where only the {[Ru(phen)_3_]^2+^} component of the RupNDIp dyad absorbs does not lead to the formation of the radical anion, Figure S30. Such results are not unexpected since excitation at 450 nm leads only to the population of the ^1^MLCT. Furthermore, the dyad is not photoactive in acetonitrile solution, regardless of the irradiation wavelength, Figures S31 and S32.

## Conclusions

The present study demonstrates that a thermal equilibrium between a fast photoinduced charge separation (CS) and a slower charge-separated state (CSS) may be established by using a compact dyad with a vibrational structure containing a long-lived triplet electron donor and a short acceptor. This provides enough time for thermal equilibrium to be reached between the ^3^CS and ^3^CSS.

## Supporting Information Available

Synthetic procedures, characterization data for all of the compounds, including NMR and mass spectra, DFT calculation, supplementary spectroscopic data, and electrochemical studies (PDF)
