# Supplementary material for: Exploring the Reversible Equilibrium State between 3CS and 3CSS in a Ru(phen)–Naphthalene Diimide Dyad
Source: Inorg Chem. 2025 Apr 21;64(17):8544–53. doi: 10.1021/acs.inorgchem.4c05443 (PMC12056696; doi:10.1021/acs.inorgchem.4c05443)
Supplement: Supplementary file 1 — ic4c05443_si_001.pdf [file ic4c05443_si_001.pdf]

Supporting Information

Exploring the reversible Equilibrium State between ^{3}CS and ^{3}CSS in a Ru(phen)-naphthalene diimide dyad

Lorena Maria Borges Pereira[†], Diego França de Oliveira[§], Marco Antonio Tiburcio[†], Gabriel H. Ribeiro[†], Carlos André Ferreira Moraes[†], Flávio Olimpio Sanches Neto [⊥, χ], Ademir João Camargo [⊥]; Leonardo De Boni [§], Otaciro Rangel Nascimento[§], Manoel G. P. Homem[†], Rose Maria Carlos[†*]

[†] Departamento de Química, Universidade Federal de São Carlos, CP 676, CEP 13565-905, São Carlos-SP, Brazil

[§] Instituto de Física de São Carlos, Universidade Estadual de São Paulo, CP 369, CEP 13560-970, São Carlos-SP, Bra-zil

[⊥] Grupo de Química Teórica e Estrutural de Anápolis, Centro de Pesquisa e Pós-Graduação, Universidade Estadual de Goiás, CP 459, CEP 75132-40, Anápolis-GO, Brazil

[χ] Instituto Federal de Educação, Ciência e Tecnologia de Goiás, CEP 72876-601, Goiânia-GO, Brazil

* *corresponding authors (rosem@ufscar.br)*

Table of Contents

1. General Methods S3

2. Synthetic Protocols and Characterization Details S4

2.1. N,N'-di(1,10-phenanthroline)-1,4,5,8-naphthalenetetracarboxylic diimide......................S4

2.2. RupNDIp dyad...S9

4. Spectroscopic Studies...S23

5. Electrochemical Properties...S25

6. Transient Absorption Spectra...S27

7. Thermodynamic Parameters...S31

8. Photoactivation of Singlet oxygen ($^1O_2$) S33

9. Photochemical Studies S35

References S37

1. General Methods

Mass spectrometric measurements were performed in positive ionization mode using an Agilent 6545 qTOF MS mass spectrometer (Agilent Technologies, Santa Clara, CA, USA) equipped with an electrospray ionization (ESI) interface. The pNDIp compound and RupNDIp dyad samples were dissolved in 100 µL of DMSO (HPLC grade) and 900 µL of MeOH (LC-MS grade), then subsequently diluted to a concentration of 5 ppm using MeOH (LC-MS grade). The mass error (in ppm) was calculated based on the theoretical mass and the corresponding MS spectrum acquired by monitoring a range of 100–1000 Da, followed by a range of 400–800 Da, with a scan rate of 3 spectra per second. Data acquisition and processing were carried out using the Mass Hunter Workstation Software version B.08.00.

The one-dimensional (^{1}H) and two-dimensional NMR measurements (^{1}H-^{1}H COSY, ^{1}H-^{13}C HSQC and ^{1}H -^{13}C HMBC) were carried out in a DMSO-d6 solution using a Bruker Advance 600 MHz spectrometer.

The electronic properties of the $[Ru(phen)_2(pNDIp)]^{2+}$ dyad were investigated using Gaussian.[1] Geometry optimizations were carried out within the framework of density functional theory (DFT), employing the hybrid B3LYP functional combined with the LANL2DZ pseudopotential for ruthenium and its ligands – this combination proved to be the most reliable after an extensive benchmark study. Solvent effects were modeled using the polarizable continuum model (PCM) for dimethyl sulfoxide (DMSO), ensuring a realistic simulation of the solution-phase environment. Time-dependent DFT (TD-DFT) calculations were conducted to evaluate the electronic excitation energies, considering 40 excited states. The analysis of hole and electron distributions was performed using the Multiwfn software[2], providing detailed insights into the electronic transitions and their spatial characteristics.

2. Synthetic Protocols and Characterization Details

2.1. N,N'-di(1,10-phenanthroline)-1,4,5,8-naphthalenetetracarboxylic diimide

The N,N'-di(1,10-phenanthroline)-1,4,5,8-naphthalenetetracarboxylic diimide (pNDIp) compound was obtained by an experimental procedure adapted from the literature. [3]For the synthesis, 1,4,5,8-naphthalenetetracarboxylic dianhydride (0.373 mmol, 100 mg), 1,10-phenanthrolin-5-amine (1.15 mmol, 225 mg) and triethylamine (Et_3N) (1.5 mmol, 215 μL) were added to 5 mL of previously distilled and deaerated dimethylformamide (DMF). The reaction mixture remained under reflux in a nitrogen atmosphere, stirring and heating at 130 °C for 3 hours. Then, the reaction mixture was cooled to room temperature. The product obtained was filtered and purified with hot DMF and washed with ethanol obtaining a yellow solid (95.7 mg, 41%) obtained in yield. MALDI-TOF MS *m/z* for $C_{38}H_{18}N_6O_4$; calcd: 622.1390; found 623.1462 (M+H)+ and 645.1296 (M+Na)+.

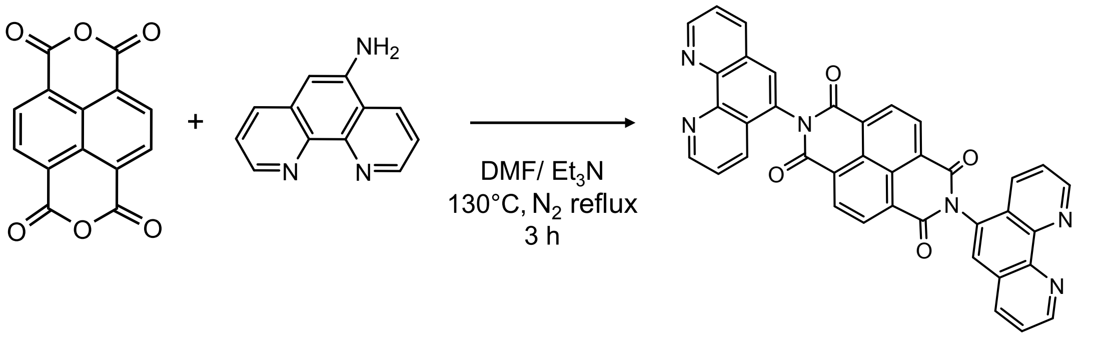

Scheme S1. Synthetic route to pNDIp compound.

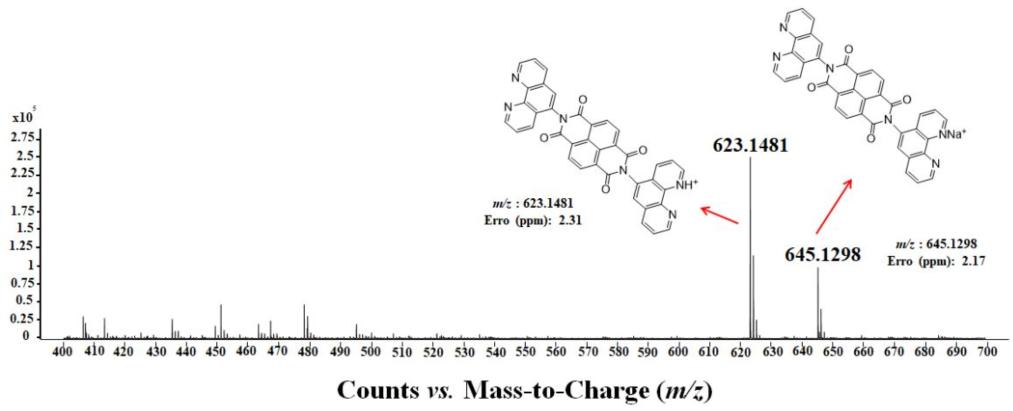

Figure S1. ESI(+)-MS spectrum of pNDIp compound in methanol.

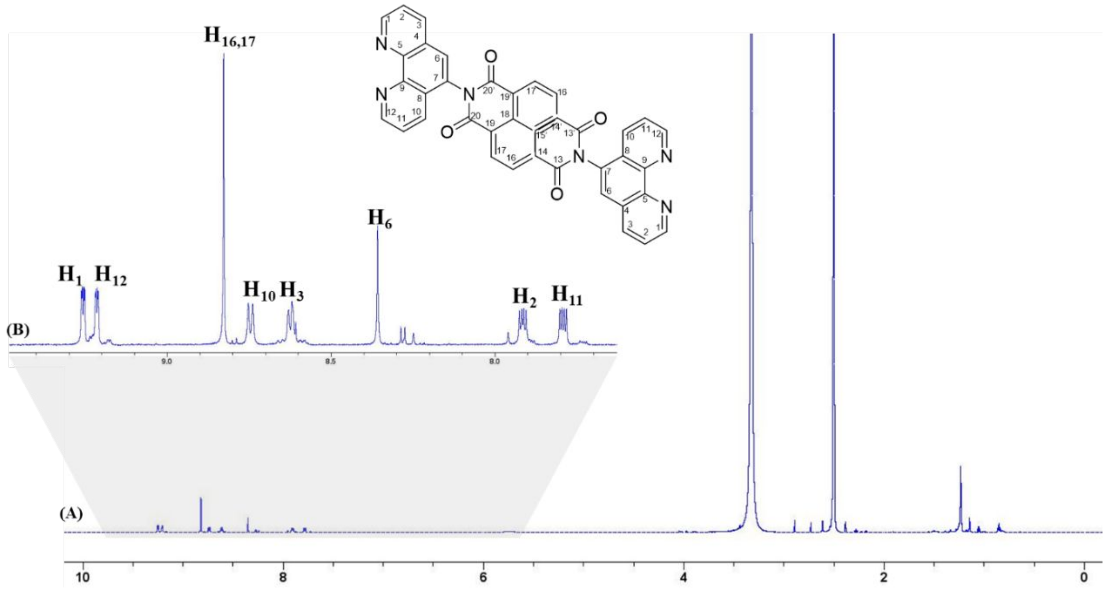

Figure S2. (A) ^{1}H NMR spectrum of pNDIp compound in DMSO-d_6 at 298K, with insertion (B) of the deshielding region of the spectrum.

Table S1. Assignments of the ^1H and $^{13}C\{^1H\}$ NMR (*via* HSQC) spectra and heternonuclear correlation data of COSY ^1H - ^1H, HSQC ^1H - ^{13}C and the main of the ^{1} H - ^{13}C HMBC in DMSO-d_6 for pNDIp compound. Chemical shifts (in ppm), multiplicity, coupling constants (Hz) and integral for hydrogens.

Moieties	No.	^{13}C via HSQC	^1H	^1H-^{13}C HMBC
pNDIp	N1			
	C1/H1	151.0	9.25 dd (1.59, 4.33) 2H	
	C2/H2	123.7	7.91 dd (4.33, 7.95) 2H	
	C3/H3	136.3	8.60 – 8.63 m 2H	
	C4	127.7	– –	7.91 (H2')
	C5	145.5	– –	8.62 (H3')
	C6/H6	127.5	8.35 s 2H	
	C7	Nd	– –	
	C8	126.3	– –	7.78 (H11')
	C9	146.1	– –	8.74 (H10')
	C10/H10	132.0	8.74 db (8.30) 2H	
	C11/H11	123.4	7.78 dd (4.15, 8.30) 2H	
	C12/H12	150.4	9.21 dd (1.51, 4.15) 2H	
	N2			
	C20/C20'; C13/C13'(C=O)	163.3		8.82 (H17, H16)
	C19/C19'; C14/C14'	127.4		8.82 (H17, H16)
	C18; C15	Nd		
	C17/H17; C16/H16	130.4	8.82 m 2H	

dd – double dublet, m – multiplet, s – singlet, db – double braod, Nd- not determined.

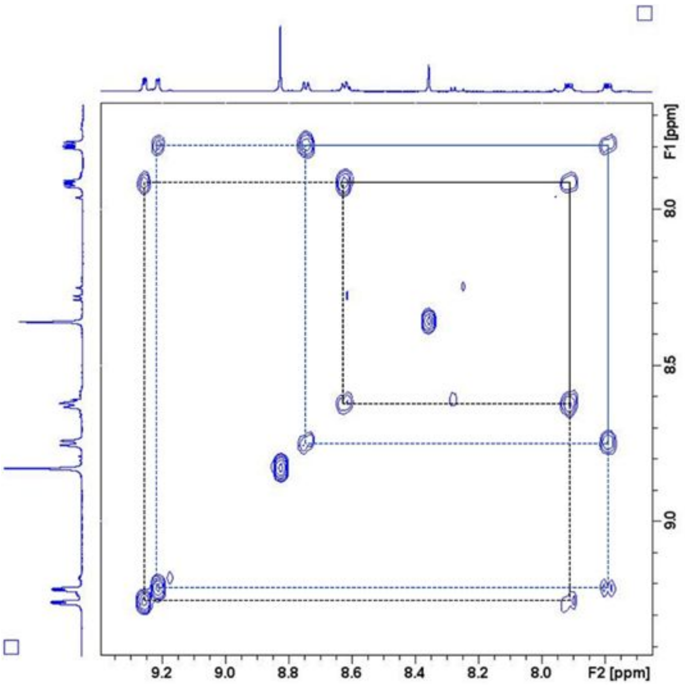

Figure S3. ^1H – ^1H COSY NMR contour map in the deshielding region of pNDIp in DMSO-d_6 at 298K.

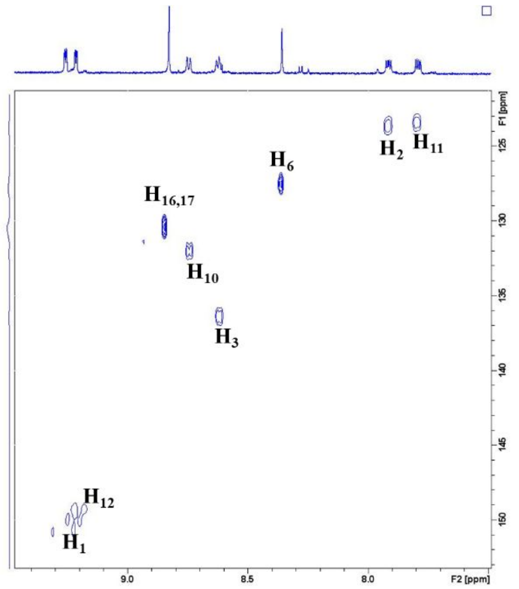

Figure S4. ^1H – $^{13}C\{^1H\}$ HSCQ NMR contour map in the deshielding region of pNDIp in DMSO-d_6 at 298K.

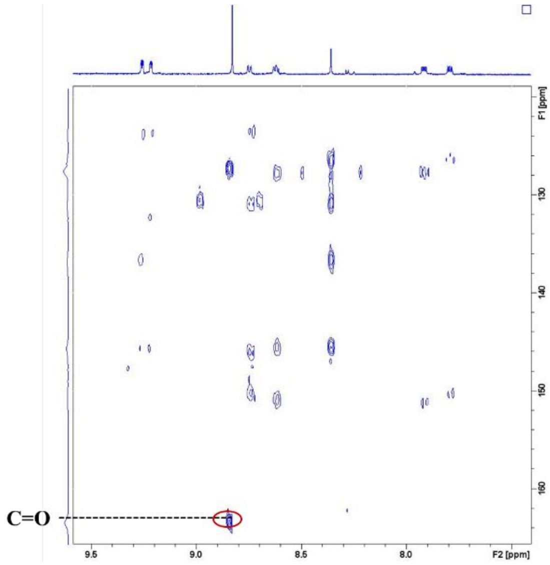

Figure S5.^{1}H – $^{13}C\{^{1}H\}$ HMBC NMR contour map in the deshielding region of pNDIp in DMSO-d_6 at 298K.

2.2. RupNDIp dyad

The dyad [Ru(phen)$_2$(pNDIp)](PF$_6$)$_2$ was synthesized from the precursor complex *cis*-[Ru(phen)$_2$Cl$_2$] (0.1 g, 0.18 mmol) and the compound pNDIp (0.17 g, 0.25 mmol) dissolved in 14 mL of previously deaerated DMF. The reaction was maintained under reflux with continuous stirring and an argon atmosphere for 24 h. After cooling to room temperature, the reaction mixture was filtered and ammonium hexafluorophosphate NH_4PF_6 (0.062 g, 0.374 mmol) was added. Then, ethyl ether was introduced to precipitate the solid product. The solid was successively recrystallized in acetone, yielding a dark red solid (0.170 g, 66 % yield). MALDI-TOF MS *m/z* for $C_{62}H_{34}N_{10}O_4RuP_2F_{12}$; calcd: 622.1390; found 1084.1798 $(M)^{2+}$.

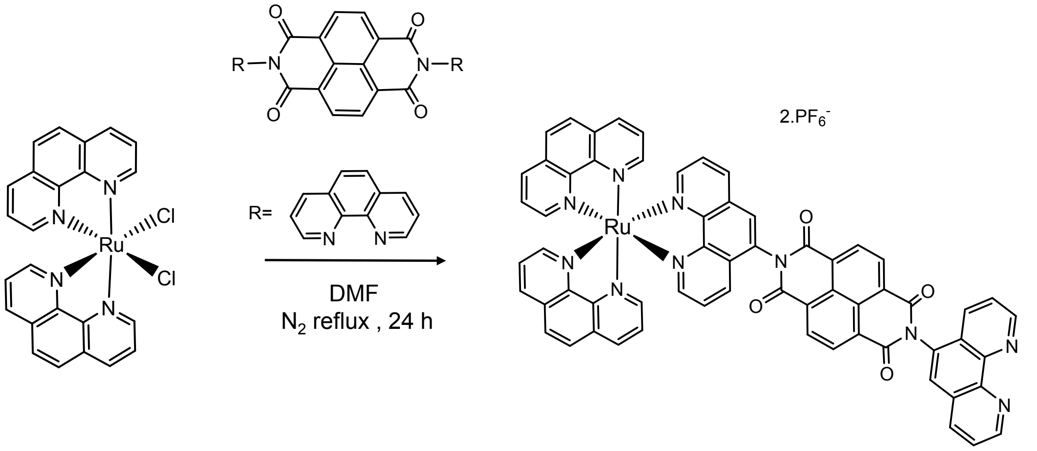

Scheme S1. Synthetic route to RupNDIp dyad.

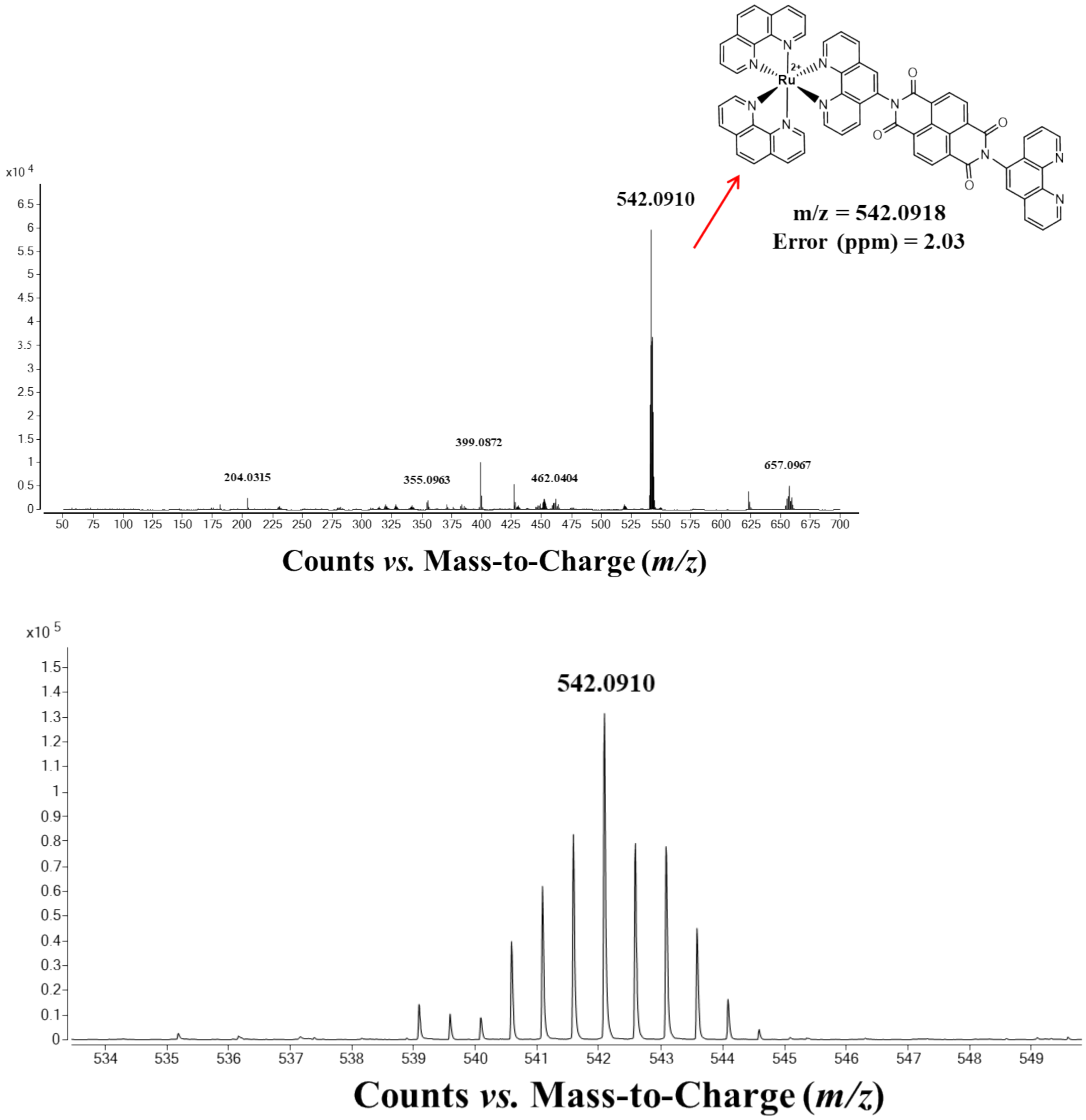

Figure S6. ESI(+)-MS spectrum of RupNDIp dyad in acetonitrile (top) and spectrum expansion on $[M]^{2+}$ ion, *m/z* =542.0910 highlighting Ruthenium isotopic distribution (bottom).

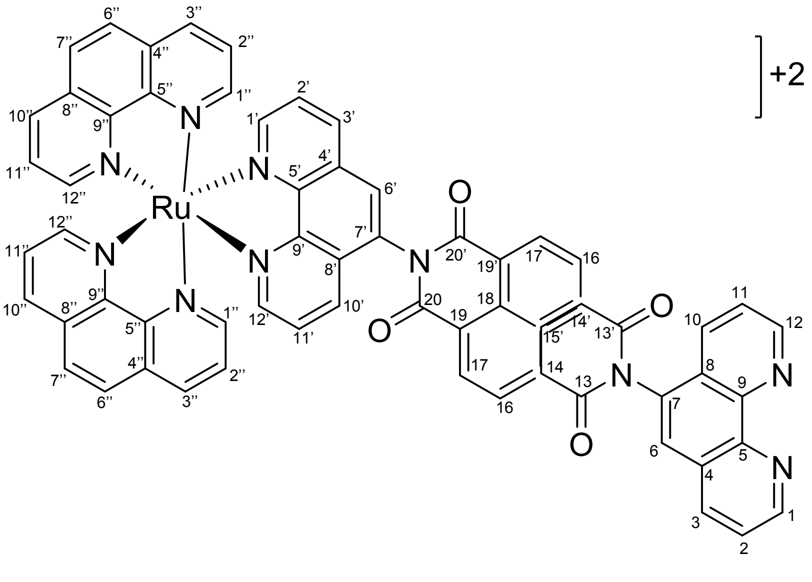

Scheme 3. Structure of the numbering pattern of the dyad for NMR assignments.

Table S2. Assignments of the ^{1}H and $^{13}C\{^{1}H\}$ NMR spectra and heteronuclear correlation data of COSY ^{1}H - ^{1}H, HSQC ^{1}H - ^{13}C, and the main of the ^{1}H - ^{13}C HMBC in DMSO-d_6 for $[Ru(phen)_2(pNDIp)]^{+2}$ dyad. Chemical shifts (in ppm), multiplicity, coupling constants (Hz) and integral for hydrogens.

Moieties	No.	^{13}C via HSQC	^{1}H	^{1}H-^{13}C HMBC
Phen	N1			
	C1''/H1''	137.1	8.81 – 8.84 m 2H	
	C2''/H2''	126.5	7.77 – 7.83 m 2H	
	C3''/H3''	153.0	8.08 – 8.15 m 2H	
	C4''	130.2 – 131.6	– –	
	C5''	147.7	– –	8.13 (H3'')
	C6''/H6''	128.5	8.40 – 8.44 m 2H	
	C7''/H7''	128.5	8.40 – 8.44 m 2H	
	C8''	130.2 – 131.6	– –	
	C9''	147.7	– –	8.21 (H12'')
	C10''/H10''	137.3	8.83 – 8.89 m 2H	
	C11''/H11''	126.8	7.84 – 7.89 m 2H	
	C12''/H12''	152.8	8.17 – 8.23 m 2H	
	N2			
pNDIp (coordinated phen moiety)	N1			
	C1'/H1'	133.5	8.97 – 9.02 m 1H	
	C2'/H2'	126.7	7.71 – 7.78 m 1H	
	C3'/H3'	153.9	8.18 – 8.22 m 1H	
	C4'	130.2 – 131.6	– –	
	C5'	147.70	– –	8.21 (H3')
	C6'/H6'	128.2	8.40 – 8.44 m 1H	
	C7'	Nd	– –	
	C8'	130.2 – 131.6	– –	
	C9'	147.7	– –	8.25 (H12')
	C10'/H10'	137.3	8.76 – 8.81 m 1H	
	C11'/H11'	127.1	7.89 – 7.95 m 1H	
	C12'/H12'	153.0	8.23 – 8.27 m 1H	
	N2			
	C20/C20' (C=O)	163,9		8.19 (H12'), 7.70 (H2')
	C19/C19'	Nd		
	C18	Nd		
	C17/H17	130.8	8.77 – 8.81 m 2H	
	C16/H16	130.8	8.80 – 8.88 m 2H	
	C15	Nd		
	C14/C14'	Nd	– –	
	C13/C13' (C=O)	163.8	– –	8.85 (H16; H16')
(non coordinated phen moiety)	N1			
	C1/H1	150.9	9.22 – 9.26 m 1H	
	C2/H2	124.3	7.90 – 7.96 m 1H	
	C3/H3	137.5	8.62 – 8.67 m 1H	
	C4	130.2 – 131.6	– –	
	C5	145.2	– –	8. 65 (H3); 9.25 (H1)
	C6/H6	128.1	8.34 – 8.39 m 1H	
	C7	Nd	– –	
	C8	130.2 – 131.6	– –	
	C9	145.1	– –	8.36 (H6); 9.20 (H10)
	C10/H10	150.5	9.19 – 9.22 m 1H	
	C11/H11	124.1	7.80 – 7.84 m 1H	
	C12/H12	129.5	8.71 – 8.77 m 1H	
	N2			

m – multiplet, Nd- not determined.

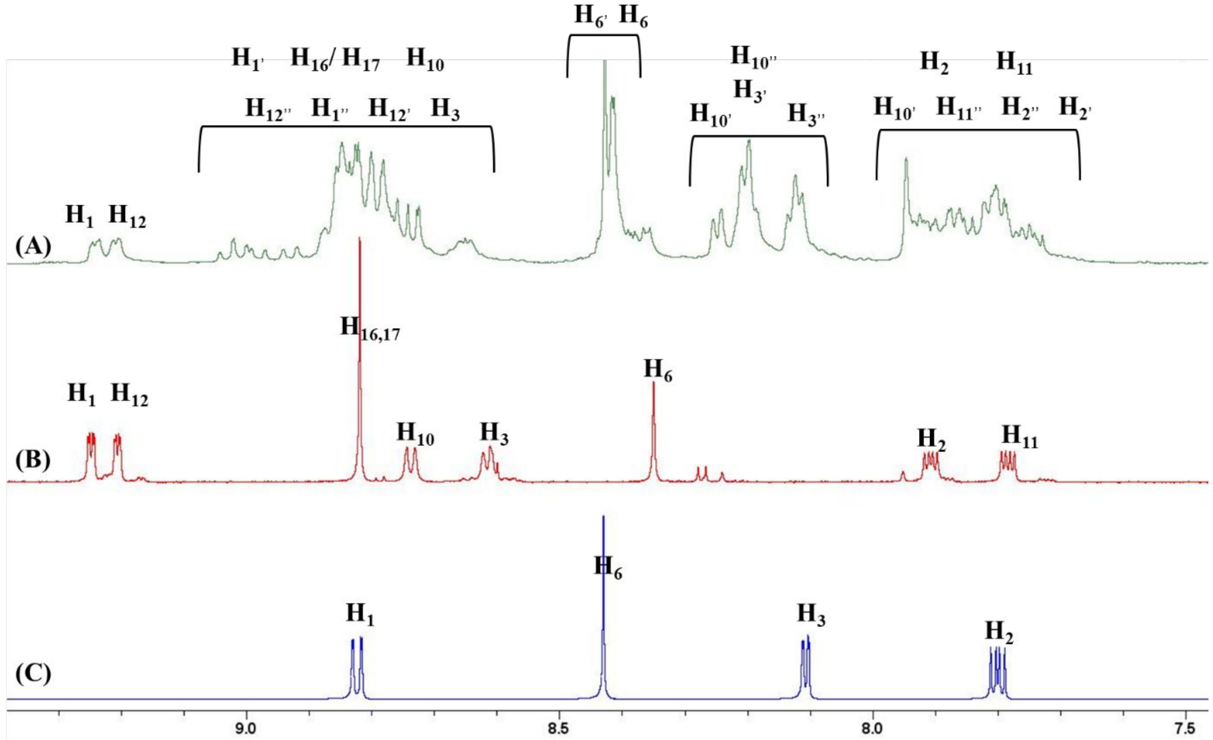

Figure S7. Deshielding region of the ^{1}H NMR spectra of (A)$[Ru(phen)_2(pNDIp)](PF_6)_2$ dyad, (B) pNDIp compound and (C) $[Ru(phen)_3]^{2+}$ complex in DMSO-d_6 at 298K.

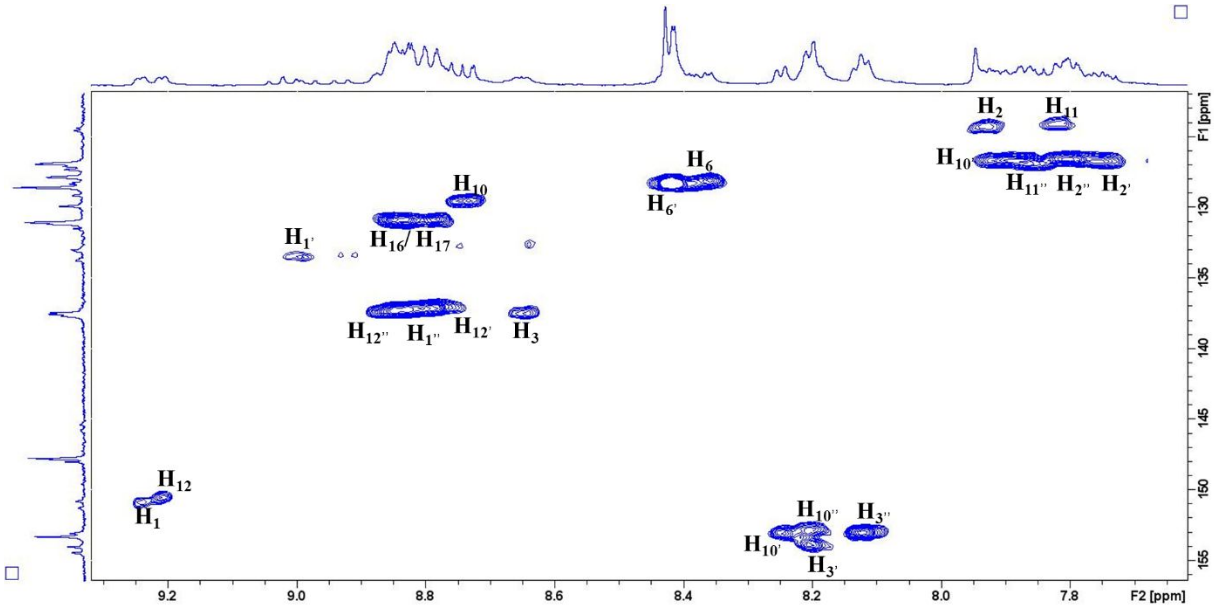

Figure S8. $^{1}H - ^{13}C\{^{1}H\}$ HSCQ NMR contour map in the deshielding region of $[Ru(phen)_2(pNDIp)](PF_6)_2$ dyad in DMSO-d$_6$ at 298K.

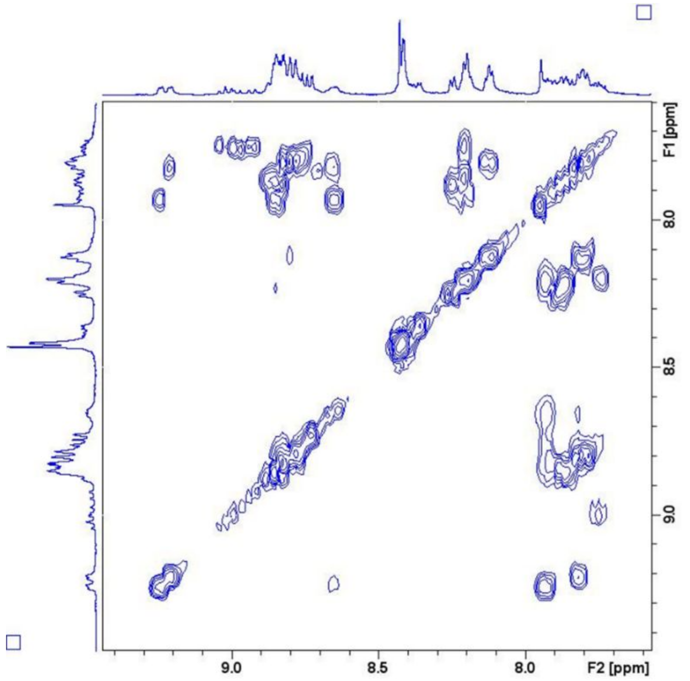

Figure S9. $^{1}H - ^{1}H$ COSY NMR contour map in the deshielding region of $[Ru(phen)_2(pNDIp)](PF_6)_2$ dyad in DMSO-d$_6$ at 298K.

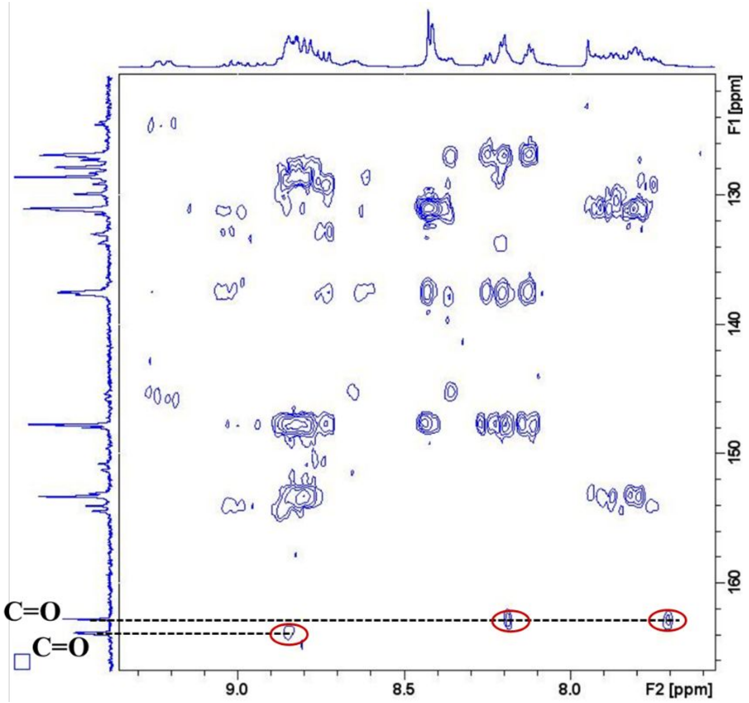

Figure S10.^{1}H – ^{13}C{^{1}H} HMBC NMR contour map in the deshielding region of $[Ru(phen)_2(pNDIp)](PF_6)_2$ dyad in DMSO-d_6 at 298K.

3. Molecular Orbital Analysis

The photophysical properties of the RupNDIp dyad and the pNDIp component were investigated using DFT at the B3LYP level with the LanL2DZ basis set, employing the Gaussian 16 program.[1] Geometries were optimized using the Polarizable Continuum Model (PCM) with dimethyl sulfoxide (DMSO) as the solvent. The optimized geometries predict that both pNDIp compound and RupNDIp dyad adopt a highly distorted geometry, leading to dihedral angles between the phen ligand and NDI of approximately 89.1°, indicating an orthogonal arrangement (Figure S11A). The distance between Ru(II) and pNDIp of 10 Å suggests weak electronic coupling, confirming our experimental results.

The primary effect of coordinating pNDIp to the $[Ru(phen)_3]^{2+}$ dyad was an increase of the HOMO orbital energy of RupNDIp by 0.79 eV and a stabilization of the LUMO orbitals by 0.07 eV compared to free NDI, as observed from the Kohn–Sham (canonical) orbitals. The decrease of the HOMO-LUMO energy gap by 0.87 eV enhances electron transfer from the Ru electron donor to the NDI electron acceptor during photoexcitation (Figure S10).

Figure S11B presents the UV-Vis absorption spectra of RupNDIp predicted by TD-DFT and compares them with the experimental spectrum in DMSO. The calculated spectra agree with the experimental data, with oscillator strengths and assignments provided in Table S2. Since multiple transitions contribute to the excited states with similar percentage contributions, examining the canonical orbitals is not particularly informative for assignment purposes. Therefore, we performed an analysis of the Natural Transition Orbitals (NTOs) to gain better insights into the electronic transitions. The assignments were made based on the NTOs, which provide a compact representation of the excitation processes, and the corresponding hole and particle orbitals are shown in Figure S11C.

The smaller oscillator strengths reflect the orthogonal structure of the dyad. Major absorption bands in the 20,000 and 33,000 cm^{-1} regions correspond to MLCT involving Ru $d\pi \rightarrow \pi^*$ transitions in the phen ligand and π–π^* transitions of pNDIp. Notably, the higher energy band at 390 nm corresponds to the $S_0 \rightarrow S_{27}$ transition, attributed to internal ligand charge transfer (ILCT) within the pNDIp component, while the strongest visible band at 430 nm corresponds to the $S_0 \rightarrow S_{17}$ transition, attributed to MLCT from Ru(II) to phen.

To gain deeper insights into the electron transfer characteristics within the RupNDIp dyad, we performed hole-electron analysis on key excited states using Multiwfn 3.8. The analysis focused on excited states S_{11}, S_{17}, S_{27}, S_{34}, S_{35}, and S_{36}, which exhibited significant transitions crucial for understanding electron dynamics. The detailed indices and parameters from the hole-electron analysis are provided in Table S3. The hole-electron analysis revealed that states S_{11} and S_{17}, corresponding to absorptions at 448.8 nm and 430 nm, respectively, involve significant MLCT character with localized excitation (LE) nature, as indicated by small electron-hole distances (D indices of 1.02 Å and 2.09 Å). State S_{27}, corresponding to the 390 nm band, shows strong LE characteristics within the pNDIp, with minimal spatial separation between the hole and electron (D index of 0.31 Å), confirming its ILCT nature.

Furthermore, states S_{34}, S_{35}, and S_{36}, corresponding to absorptions around 350 nm, exhibit varying degrees of charge transfer characteristics involving both π–π* and MLCT transitions. State S_{34} has a D index of 3.70 Å, indicative of single-direction charge transfer involving both π–π* and MLCT transitions with a minor pNDIp contribution. States S_{35} and S_{36} show significant charge transfer from Ru(II) to the pNDIp ligand, as evidenced by large D indices (8.04 Å and 8.48 Å), consistent with MLCT transitions.

These electronic excitations align with the irradiation wavelengths studied at 450 nm and 350 nm. The data indicate that excitation at 450 nm preserves the low-energy absorption of the $[Ru(phen)_3]^{2+}$ chromophore, while excitation at 350 nm promotes interconversion among excited states involving both pNDIp and $\{[Ru(phen)_3]^{2+}\}$ components.

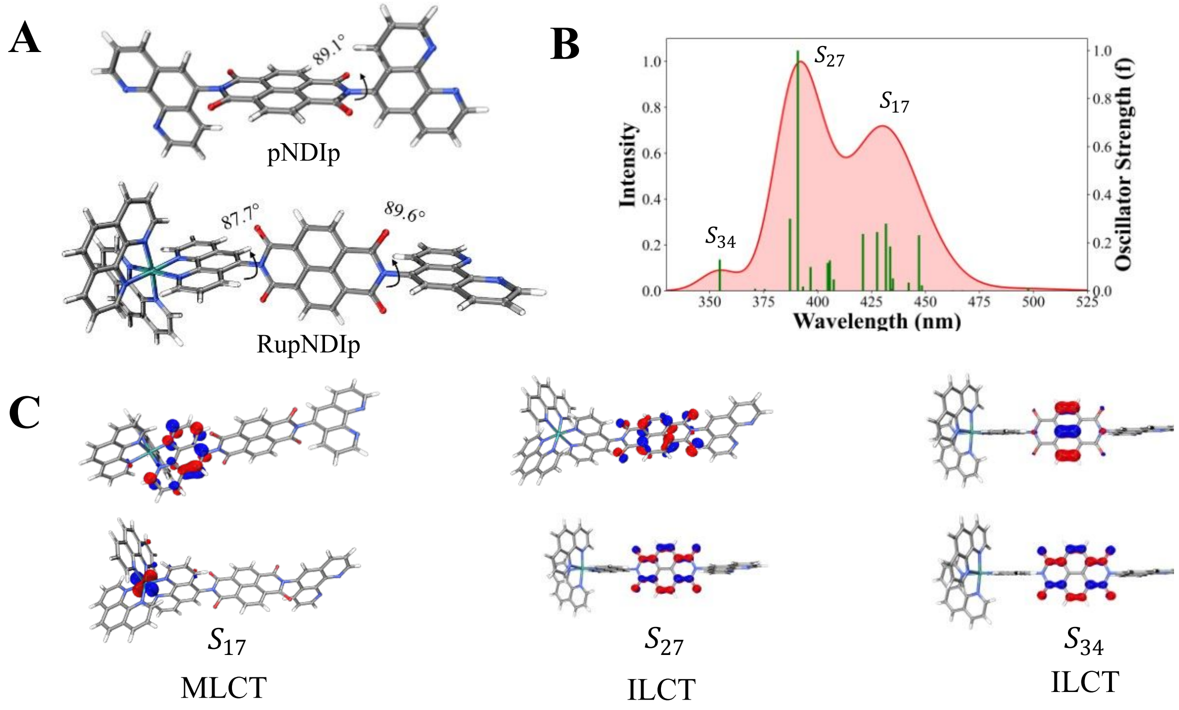

Figure S11. Computational results for the ground state and absorption properties of the RupNDIp dyad. (A) DFT-optimized structure with key torsion angles. (B) TDDFT absorption spectrum with highlighted singlet-singlet transitions and oscillator strengths. (C) Selected electronic transitions with assignments based on the NTOs.

Table S3: Key indices from hole-electron analysis of excited states in RupNDIp complexes using Multiwfn.

Excited State	Wavelength (nm)	oscillator strengths	D (Å)	S_r (a.u)	t (Å)	$\Delta\sigma$ (Å)	Δr	Assignment	MLCT(%)
$S_0 \rightarrow S_1$	675.7	0.00010	9.99	0.019	8.302	1.432	10.121	$d_{Ru} - \pi^*_{NDI}$	84.68
$S_0 \rightarrow S_2$	628.1	0.00120	9.74	0.035	7.848	0.927	9.975	$d_{Ru} - \pi^*_{NDI}$	74.50
$S_0 \rightarrow S_3$	624.6	0.00010	10.38	0.018	8.548	0.952	10.323	$d_{Ru} - \pi^*_{NDI}$	75.29
$S_0 \rightarrow S_4$	497.0	0.00130	2.57	0.349	1.267	1.099	2.234	$d_{Ru} - \pi^*_{phen}$	81.45
$S_0 \rightarrow S_5$	491.5	0.00390	6.39	0.111	4.344	0.590	6.674	$\pi_{phen} - \pi^*_{NDI}$	0.00
$S_0 \rightarrow S_6$	480.2	0.00010	1.26	0.445	-0.237	1.871	1.490	$d_{Ru} - \pi^*_{phen}$	79.61
$S_0 \rightarrow S_7$	478.4	0.00130	1.42	0.382	-0.064	1.863	1.045	$d_{Ru} - \pi^*_{phen}$	83.78
$S_0 \rightarrow S_8$	464.1	0.00150	2.72	0.390	1.208	0.863	2.502	$d_{Ru} - \pi^*_{phen}$	74.54
$S_0 \rightarrow S_9$	462.2	0.00010	2.75	0.394	1.046	1.579	2.508	$d_{Ru} - \pi^*_{phen}$	82.57
$S_0 \rightarrow S_{10}$	456.5	0.00010	8.43	0.051	6.616	1.061	8.605	$\pi_{phen} - \pi^*_{NDI}$	0.00
$S_0 \rightarrow S_{11}$	448.8	0.00600	1.02	0.540	-0.583	1.585	1.666	$d_{Ru} - \pi^*_{phen}$	75.32
$S_0 \rightarrow S_{12}$	446.4	0.08510	0.37	0.456	-1.498	1.813	2.041	$d_{Ru} - \pi^*_{phen}$	78.15
$S_0 \rightarrow S_{13}$	441.6	0.04450	1.20	0.589	-0.769	1.702	1.792	$d_{Ru} - \pi^*_{phen}$	72.99
$S_0 \rightarrow S_{14}$	434.9	0.00270	0.63	0.486	-1.328	2.313	1.600	$d_{Ru} - \pi^*_{phen}$	80.84
$S_0 \rightarrow S_{15}$	434.5	0.07450	1.46	0.560	-0.273	1.567	1.285	$d_{Ru} - \pi^*_{phen}$	74.84
$S_0 \rightarrow S_{16}$	432.4	0.00800	7.05	0.089	4.721	-0.039	7.279	$\pi_{phen} - \pi^*_{NDI}$	0.15
$S_0 \rightarrow S_{17}$	430.4	0.14780	2.09	0.477	-0.350	1.232	2.296	$d_{Ru} - \pi^*_{phen}$	76.61
$S_0 \rightarrow S_{18}$	426.7	0.07640	2.07	0.473	0.180	1.641	2.203	$d_{Ru} - \pi^*_{phen}$	78.23
$S_0 \rightarrow S_{19}$	422.1	0.12110	1.29	0.442	-0.361	2.242	1.644	$d_{Ru} - \pi^*_{phen}$	82.56
$S_0 \rightarrow S_{20}$	411.4	0.04000	5.49	0.339	2.872	-0.223	4.461	$\pi_{phen} - \pi^*_{NDI}$	2.93

$S_0 \rightarrow S_{21}$	407.1	0.01970	1.69	0.443	-0.322	1.876	1.254	$d_{Ru} - \pi^*_{phen}$	77.22
$S_0 \rightarrow S_{22}$	405.1	0.05000	1.76	0.406	-0.090	1.966	1.620	$d_{Ru} - \pi^*_{phen}$	77.96
$S_0 \rightarrow S_{23}$	399.6	0.00240	1.83	0.388	0.242	1.950	1.745	$d_{Ru} - \pi^*_{phen}$	78.74
$S_0 \rightarrow S_{24}$	396.9	0.04130	1.54	0.436	-0.170	1.959	1.528	$d_{Ru} - \pi^*_{phen}$	78.62
$S_0 \rightarrow S_{25}$	396.5	0.00190	11.68	0.101	9.487	-1.024	11.146	$\pi_{phen} - \pi^*_{NDI}$	2.96
$S_0 \rightarrow S_{26}$	393.2	0.00060	11.64	0.052	9.495	-0.960	11.655	$\pi_{phen} - \pi^*_{NDI}$	5.85
$S_0 \rightarrow S_{27}$	390.1	0.56550	0.31	0.838	-2.344	-0.106	2.386	$\pi_{NDI} - \pi^*_{NDI}$	0.33
$S_0 \rightarrow S_{28}$	386.9	0.00010	8.35	0.083	6.416	0.929	7.423	$\pi_{phen} - \pi^*_{NDI}$	0.00
$S_0 \rightarrow S_{29}$	384.5	0.02630	0.25	0.494	-2.547	-1.534	1.747	$\pi_{NDI} - \pi^*_{NDI}$	0.57
$S_0 \rightarrow S_{30}$	376.1	0.00280	0.32	0.597	-1.624	1.960	1.305	$d_{Ru} - \pi^*_{phen}$	74.61
$S_0 \rightarrow S_{31}$	370.7	0.00190	1.63	0.441	-1.727	-1.623	2.402	$\pi_{NDI} - \pi^*_{NDI}$	0.40
$S_0 \rightarrow S_{32}$	360.6	0.00160	6.82	0.274	4.034	-1.024	7.492	$\pi_{phen} - \pi^*_{NDI}$	1.39
$S_0 \rightarrow S_{33}$	357.7	0.00180	10.73	0.169	8.066	-1.351	10.324	$\pi_{phen} - \pi^*_{NDI}$	1.22
$S_0 \rightarrow S_{34}$	353.5	0.04330	3.70	0.582	-0.290	-3.014	3.650	$\pi_{NDI} - \pi^*_{NDI}$	0.77
$S_0 \rightarrow S_{35}$	352.3	0.01600	8.04	0.358	4.325	-2.773	7.233	$\pi_{phen} - \pi^*_{NDI}$	1.85
$S_0 \rightarrow S_{36}$	351.1	0.00030	8.48	0.264	5.935	2.551	8.664	$d_{Ru} - \pi^*_{NDI}$	77.64
$S_0 \rightarrow S_{37}$	345.2	0.00070	0.39	0.672	-1.163	0.859	1.369	$d_{Ru} - d_{Ru}$	21.74
$S_0 \rightarrow S_{38}$	344.7	0.00090	0.92	0.670	-1.295	1.825	1.820	$d_{Ru} - d_{Ru}$	26.21
$S_0 \rightarrow S_{39}$	337.3	0.00020	8.75	0.247	6.219	1.295	8.914	$d_{Ru} - \pi^*_{NDI}$	71.88
$S_0 \rightarrow S_{40}$	336.7	0.00010	0.92	0.493	-2.126	-0.976	1.547	$\pi_{NDI} - \pi^*_{NDI}$	0.62

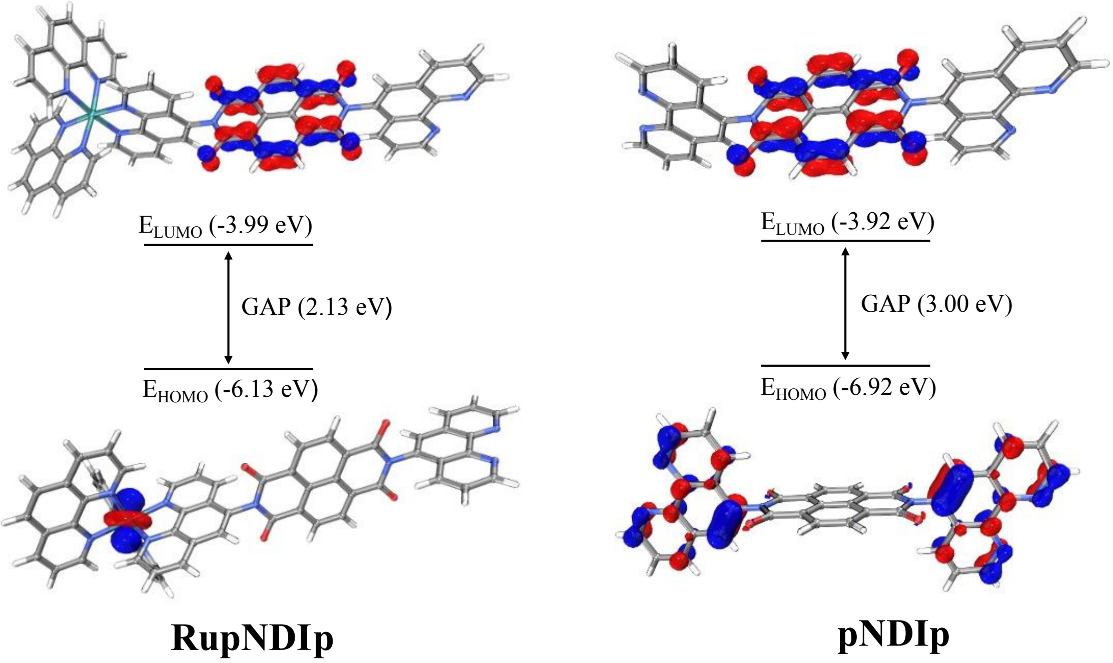

Figure S12: Highest Occupied Molecular Orbital (HOMO), Lowest Unoccupied Molecular Orbital (LUMO), and calculated HOMO-LUMO energy gap for the RupNDIp dyad and the pNDIp. The orbitals were obtained from DFT calculations at the B3LYP/LanL2DZ level of theory using the PCM model with DMSO as the solvent.

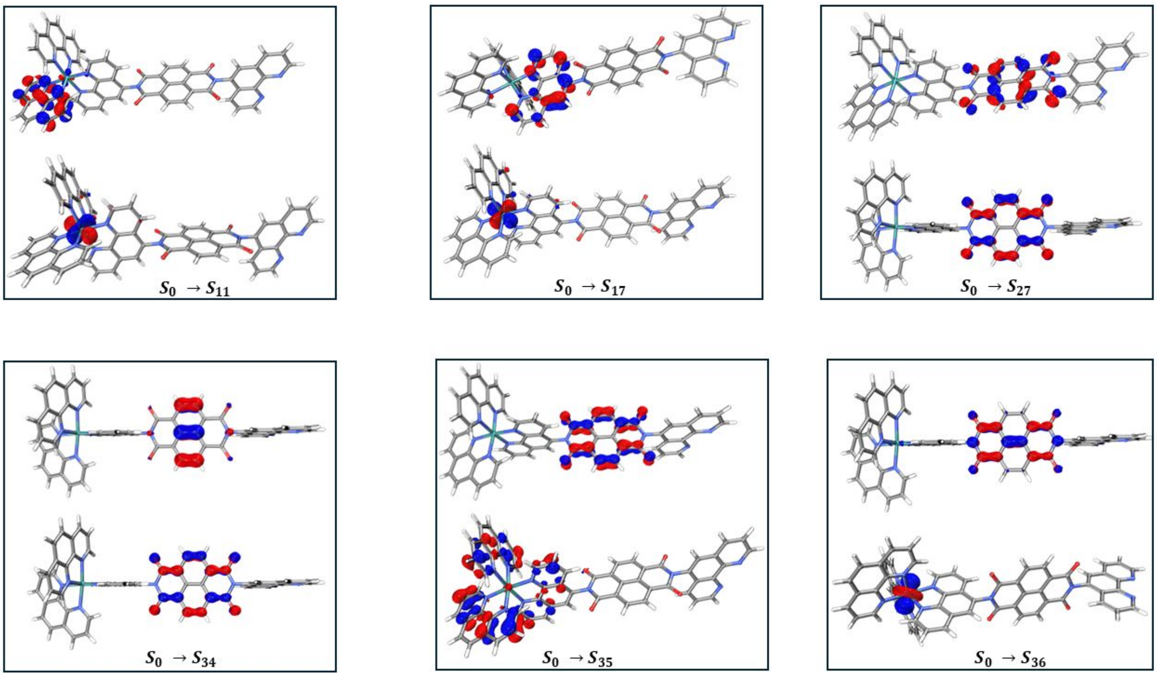

Figure S13: Natural Transition Orbitals (NTOs) for excited states S_{11}, S_{17}, S_{27}, S_{34}, S_{35}, and S_{36} of the RupNDIp dyad. The hole (HOTO) and particle (LUTO) orbitals are depicted for each transition, highlighting the electronic excitation characteristics. These NTOs were derived from TD-DFT calculations at the B3LYP/LanL2DZ level of theory using the PCM model with DMSO as the solvent.

4. Spectroscopic Studies

Table S4. Photophysical properties in DMSO solution.

Compound	λ_{Abs}, nm (ε, $M^{-1}.cm^{-1}$)	λ_{EM}, nm	τ_{EM}, ns	Φ_{EM}
RupNDIp	365 (15850)<br>385 (19000)<br>450 (15300)	610	540	0.0075
$[Ru(phen)_3]^{2+}$	450 (15000)	605	950	0.05

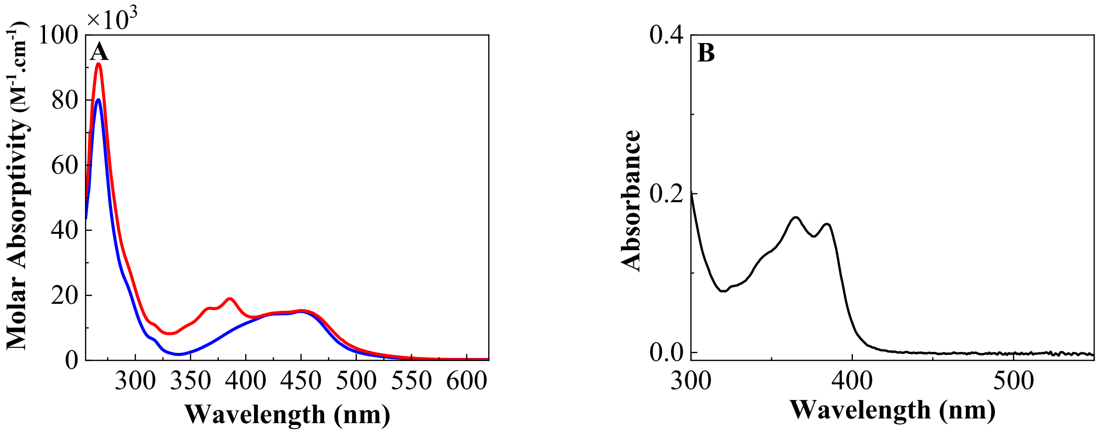

Figure S14. Absorption Spectrum of (A) $[Ru(phen)_3]^{2+}$ (blue) and RupNDIp dyad (red) in DMSO solution (c = 20 µmol.L^{-1}) and (B) pNDIp compound.

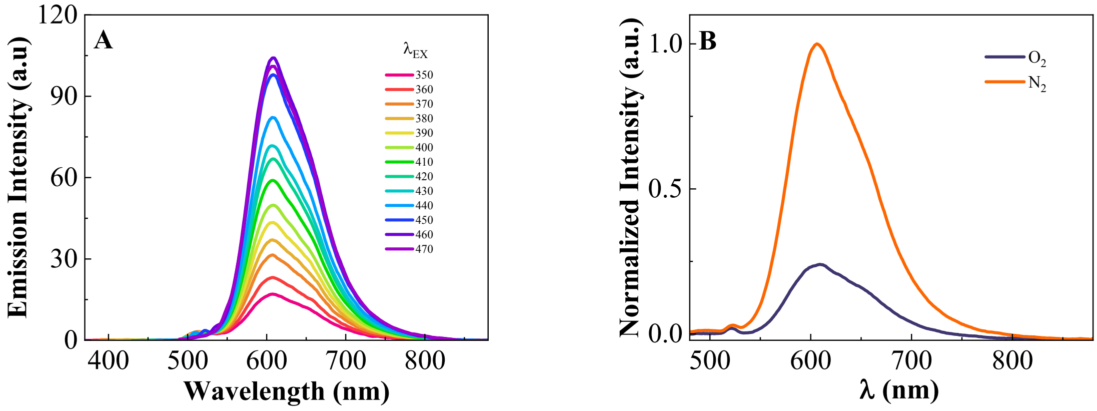

Figure S15. (A) Emission spectra of the RupNDIp dyad at different excitation wavelengths (350-470nm) and (B) Comparison of the emission spectrum of the RupNDIp dyad in aerated and deaerated solution (λ_{EX}=450 nm).

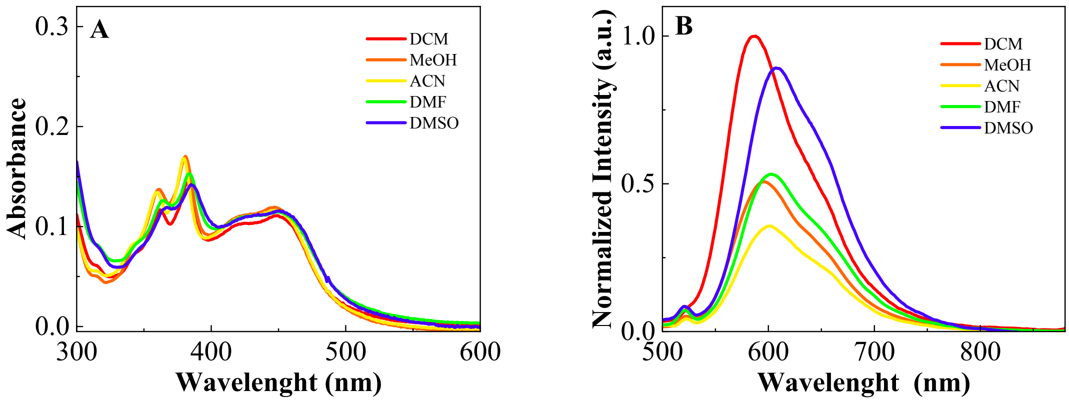

Figure S16. Absorption (A) and Emission spectra (B) (λ_{EX} = 450 nm) of RupNDIp dyad in different organic solvents at room temperature in air- equilibrated solutions.

5. Electrochemical Properties

The cyclic voltammograms of RupNDIp dyad in acetonitrile (Figure S17) exhibit a well-defined metal-based redox (RuIII/II couple at 0.635 V *versus* Fc^{+}/Fc (100 $mV.s^{-1}$), and two reversible one-electron reduction processes with half-wave potentials of – 1.06 V and −1.33 V *versus* Fc^{+}/Fc assigned to the sequential formation of the radical anions $pNDIp^{\bullet-}$ and dianion $pNDIp^{2-}$, respectively. The cyclic voltammograms of pNDIp compound and RupNDIp dyad a (Figure S18) in DMSO solution exhibit low negative values for the first reduction indicate that the pNDIp can be easily reduced to $pNDIp^{\bullet-}$. No significative change in the redox potential of the components was observed with the coordination of pNDIp to Ru(II) indicating that the electron-withdrawing nature of NDI does not affect the redox potential of dyad.

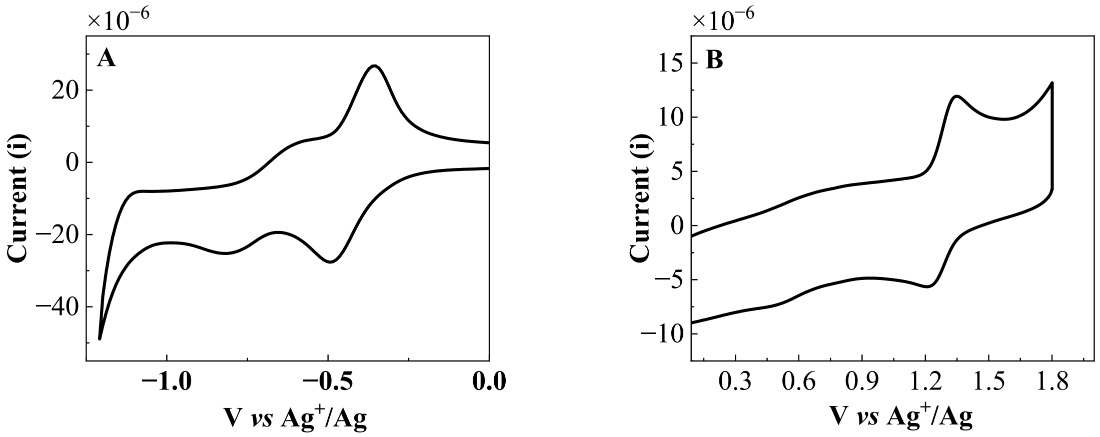

Figure S17. Cyclic voltammogram of the RupNDIp dyad in acetonitrile ($TBAPF_6$, 0.1 M) solutions, glassy carbon working electrode (V *vs* Ag wire as pseudoreference), and a scan rate of 100 mVs^{-1}. $E_{1/2}$ for Fc^{+}/Fc = +0.635V measured under the same experimental conditions. (A) reduction and (B) oxidation.

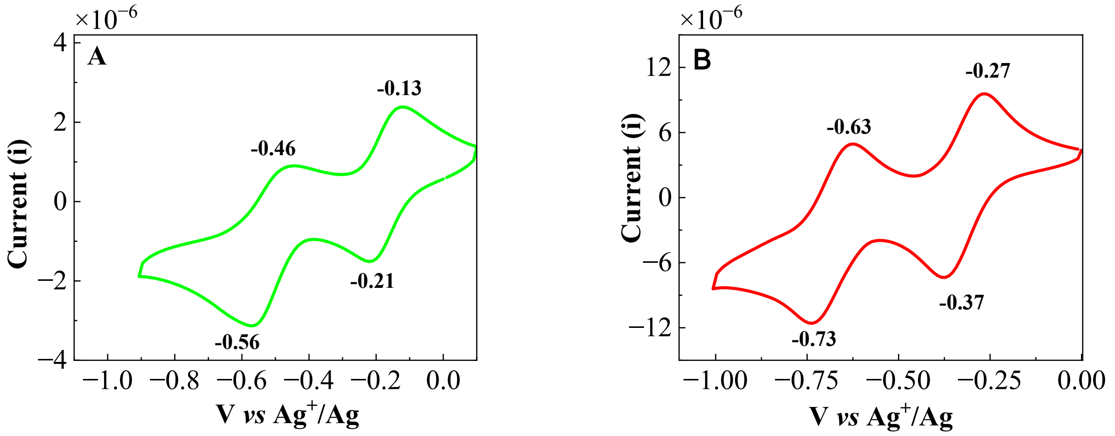

Figure S18. Cyclic voltammogram in DMSO ($TBAPF_6$, 0.1 M) solutions, glassy carbon working electrode (V *vs* Ag wire as pseudoreference), and a scan rate of 100 mVs^{-1}. $E_{1/2}$ for Fc^+/Fc = +0.72V measured under the same experimental conditions. (A) pNDIp compound and (B) RupNDIp dyad.

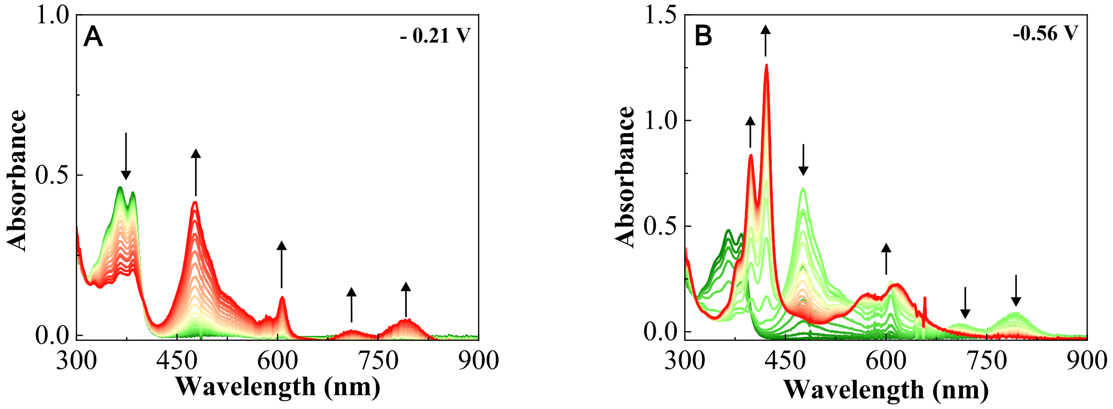

Figure S19. Spectroelectrochemical analysis for pNDIp in degassed DMSO ($TBAPF_6$, 0.1 M) at (A) E= -0.21 (V *vs* Ag wire as pseudoreference) and (B) E= -0.56 (V *vs* Ag wire as pseudoreference).

6. Transient Absorption Spectra

Pump-and-probe experimental setup used for transient absorption measurements is displayed in Scheme S4. A pulsed laser (Light Conversion PHAROS) with active medium of Yb:KGW and fundamental emission at 1030nm. The system has 6W of power, works at a repetition rate of 7.5kHz and deliver pulses of 220fs. The fundamental beam is separated by a beam splitter and used to generate two pulses: the pump, which is creates an excited state population, and a probe, which is used to observed alterations in the absorption spectrum as the excited population varies in time. The pump pulse is created using an Optical parametric Amplificator (OPA) (Light Conversion, Model ORPHEUS), tuning the wavelength of the pump beam from 220nm to 3μm. After going through the OPA, the pump beam passes through a Half-wave plate that controls the polarization of the beam. For the transient absorption measurements performed the angle between the Pump and the probe polarization is set at 54.7°, also known as the "magic angle", which ensures that the measurements are not influenced by polarization induced effects and therefore emerge due to purely electronic effects. The pump beam passes through a delay line made of a pair of mirrors that can be moved together using a translation stage. The line has a total temporal variation of 600ps with steps of 13fs of variation. The number of steps between measurements grows exponentially with time, providing higher temporal resolutions for the faster dynamics and lower resolution for slower dynamics, this optimizes the time of the measurement. The pump beam then crosses a chopper which is synchronized with the laser repetition rate and will either complete block or completely transmit the pump pulse. If the pump is block (pump-OFF configuration) the probe beam will measure a purely ground state, and if the pump is transmitted (pump-ON configuration) the probe will observe the transient excited state dynamics. The Pump reflects in a set of flat mirrors and lastly in a concave mirror, being then sent to the sample.

The probe pulse, separated in the first beam splitter, goes through a lens an is focalized in a sapphire crystal, generating a super continuum of light, which is then collimated by another lens. The probe pulse reflects on a set of flat mirrors and lastly

on the concave mirror, being delivered at the sample in the same spot as the pump beam. The size of the probe beam is controlled so that it is smaller than the pump, to ensure maximum sensitivity.

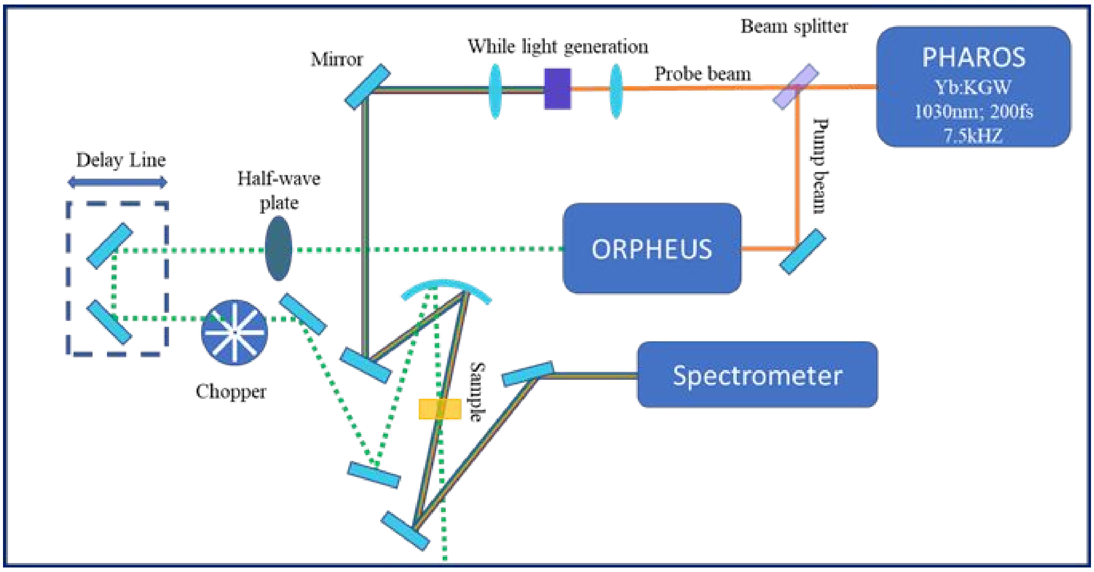

Scheme S4. Pump-and-probe experimental setup diagram.

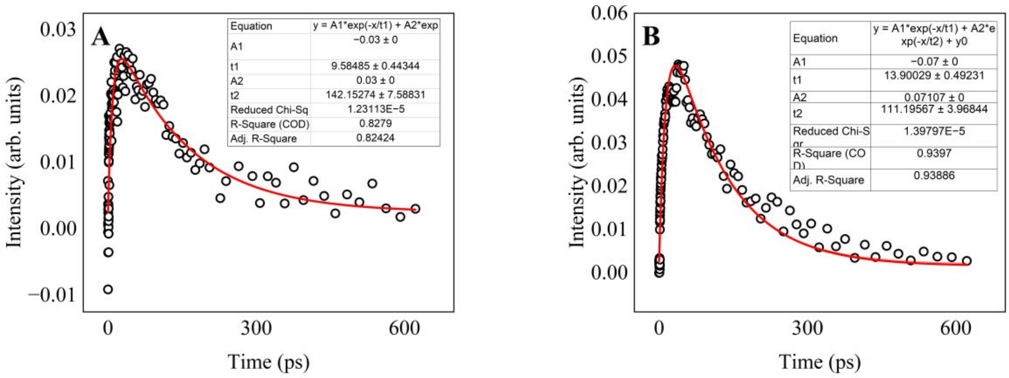

Figure S19. Transient absorption of RupNDIp dyad in (A) DMSO and (B) acetonitrile with λ_{EX} = 450 nm as a function of time. The black point represents data, and the red line represents the biexponential fitting.

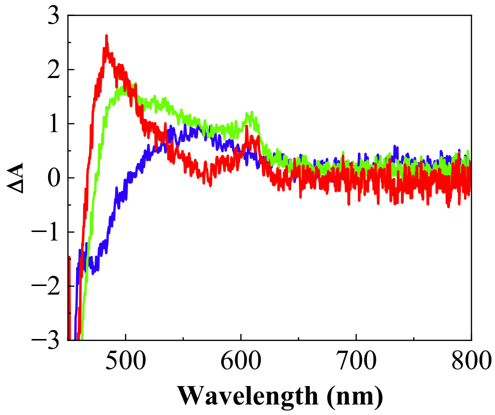

Figure S20. Transient absorption measurements of RupNDIp dyad (green line), $[Ru(phen)_3]^{2+}$ complex (blue line), and the difference between the two spectra (red line) in DMSO solution.

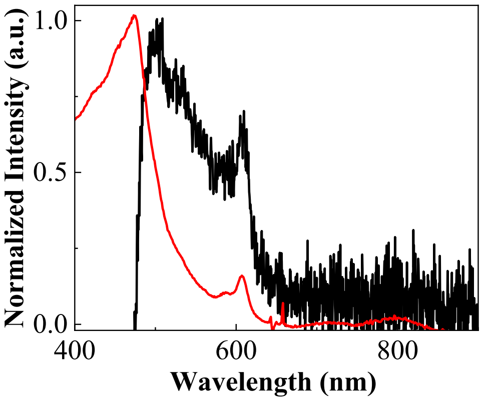

Figure S21. Comparison of the normalized femtosecond transient absorption spectrum (black line) and the spectroelectrochemical plot (red line) of the RupNDI dyad ($TBAPF_6$, 0.1 M) at applied potentials E = -0.37 V (vs Ag^+/Ag wire as pseudoreference) in DMSO solution.

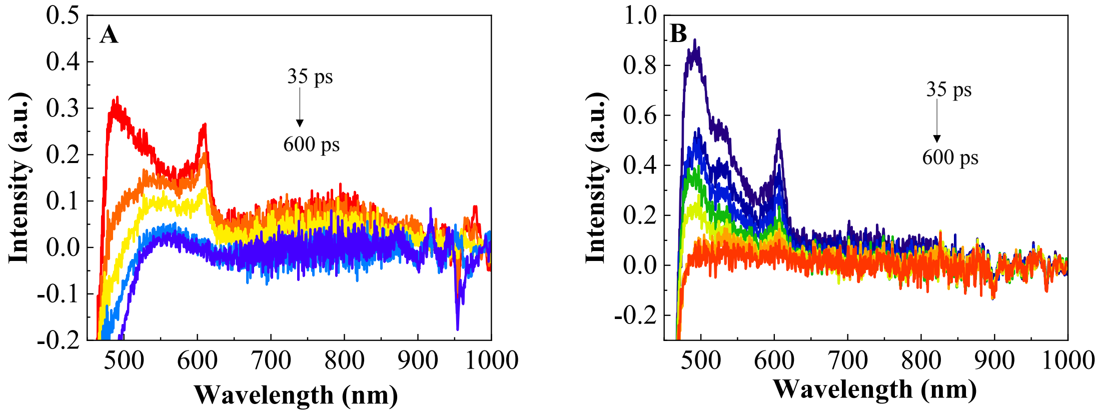

Figure S22. Transient absorption spectra of RupNDIp dyad with λ_{EX} = 450 nm for different time delays from 35 to 600 ps in (A) DMSO and (B) acetonitrile.

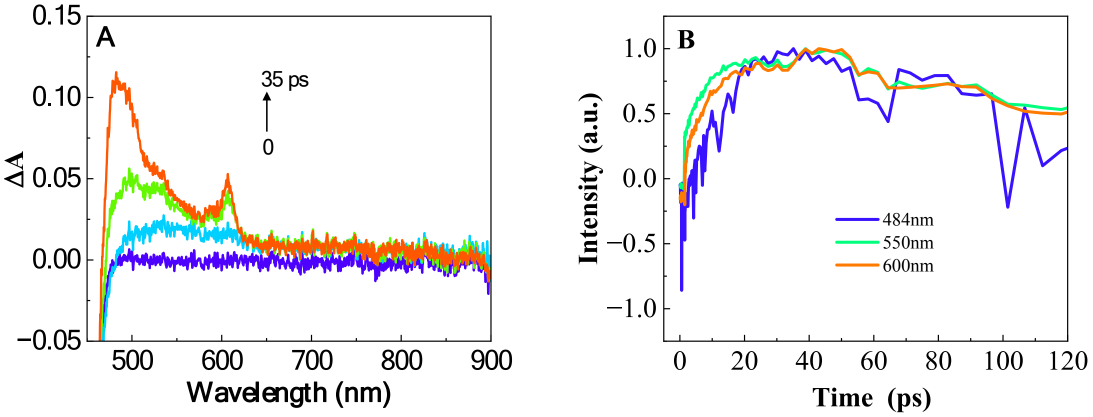

Figure S23. Transient absorption graphs of RupNDIp dyad in acetonitrile (360 μM) with λ_{EX} = 450 nm (A) Transient absorption spectra for different time delays from 0 to 35 ps and (B) Normalized transient absorption values at different wavelengths as a function of time.

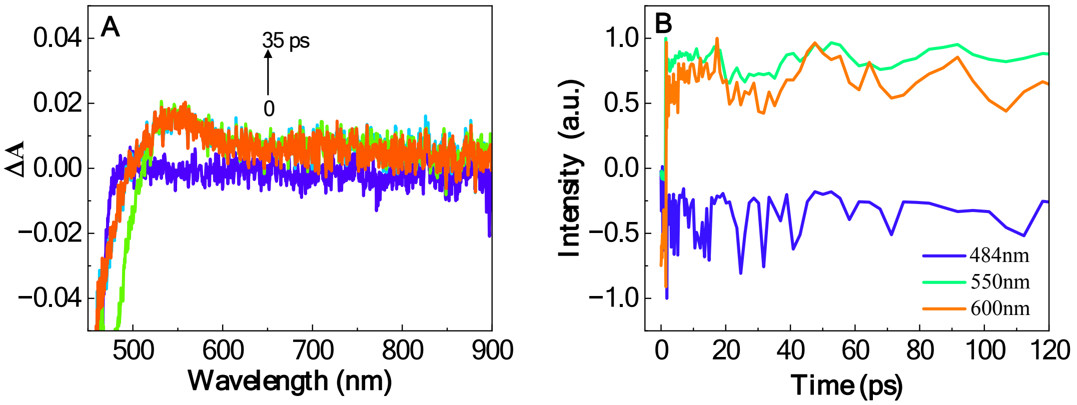

Figure S24. Transient absorption graphs of $[Ru(phen)_3]^{2+}$ complex in acetonitrile with λ_{EX} = 450 nm (A) Transient absorption spectra for different time delays and (B) Normalized transient absorption values at different wavelengths as a function of time.

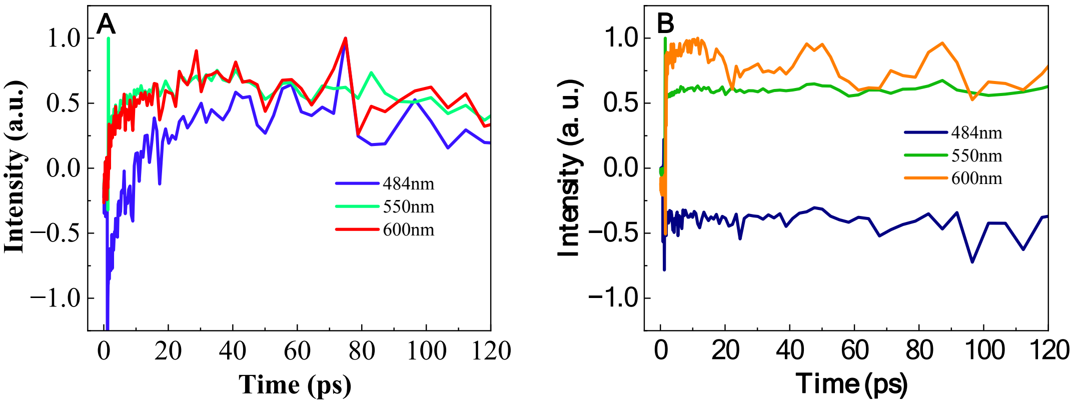

S25. Normalized transient absorption values at different wavelengths as a function of time (A) RupNDIp dyad and (B) $[Ru(phen)_3]^{2+}$ complex in DMSO solution.

7. Thermodynamic Parameters

The dynamic of photoinduced electron transfer of RupNDIp was analyzed by the Marcus Theory of electron transfer.[4] Specifically, the driving force for photoinduced electron transfer ($^{*}\Delta G_{CT}$), the reorganization energy (λ), and the electronic coupling (H_{DA}) between the $[Ru(phen)_3]^{2+}$ and pNDIp components were determined.

Due to the limited potential range of DMSO in cyclic voltammetry experiments, the Gibbs free energy change for charge separation ($^*\Delta G_{CT}$) of the RupNDIp dyad was calculated using data obtained in acetonitrile (Figure S17). This calculation employed the Rehm-Weller equation given by: [5,6]

$$^*\Delta G_{CT} = \{[E(\frac{D^+}{D}) - \Delta E_{00}] - E(\frac{A}{A^-})\} + w_p \quad (1),$$

Here, $E(\frac{D^+}{D})$ represents the oxidation potential of the donor (D), which corresponds to the energy required to remove an electron from D, converting it into its oxidized form (D^+). $E(\frac{A}{A^-})$ is the reduction potential of the acceptor (A), which reflects the energy needed to add an electron to A, converting it into its reduced form (A^-). ΔE00 represents the energy of the ^{3}MLCT state (2.05 eV), and W_p is the Coulombic energy associated with the intermediate radical ion-pair, given by:

$$w_p = (\frac{e^2}{4\pi\epsilon_0})(\frac{1}{\epsilon_s r_{DA}}) \quad (2),$$

where ϵ_s is the static dielectric constant of the solvent (ϵ_s = 36.5 for acetonitrile) and r_{DA} = 10.12Å, as determined from the DFT-optimized geometry.

The reorganization energy (λ) was calculated using the Born-Hush approach, which considers both internal (λ_i) and external (λ_o) contributions. Here, λ_i was assumed to be negligible and the λ_o obtained by:[7,8]

$$\lambda_o = (\frac{e^2}{4\pi\epsilon_0})(\frac{1}{\epsilon_{op}} - \frac{1}{\epsilon_s})(\frac{1}{2r_A} + \frac{1}{2r_D} - \frac{1}{r_{DA}}) \quad (3),$$

where r_D = 6.37Å and r_A = 7.27Å was also determined from the DFT-optimized geometry, and ϵ_{op} = 1.8 (optical dieletric constant for acetonitrile).

The electronic coupling term (H_{DA}) was evaluated from the present spectroscopic absorption data using the Mulliken-Hush relation:[9–11]

$$H_{DA} = 0.0206\,(r_{DA})^{-1}(\nu_{max}\,\epsilon_{max}\,\Delta\nu_{max})^{1/2} \quad (4),$$

where ν_{max} is the frequency of the charge transfer band maximum (in cm^{-1}), $\Delta\nu_{max}$ is the full width at half maximum (FWHM) of the charge transfer band (in cm^{-1}), and ϵ_{max} is the molar absorptivity at the band maximum (in $L.mol^{-1}.cm^{-1}$).

8. Photoactivation of Singlet oxygen ($^1O_2$)

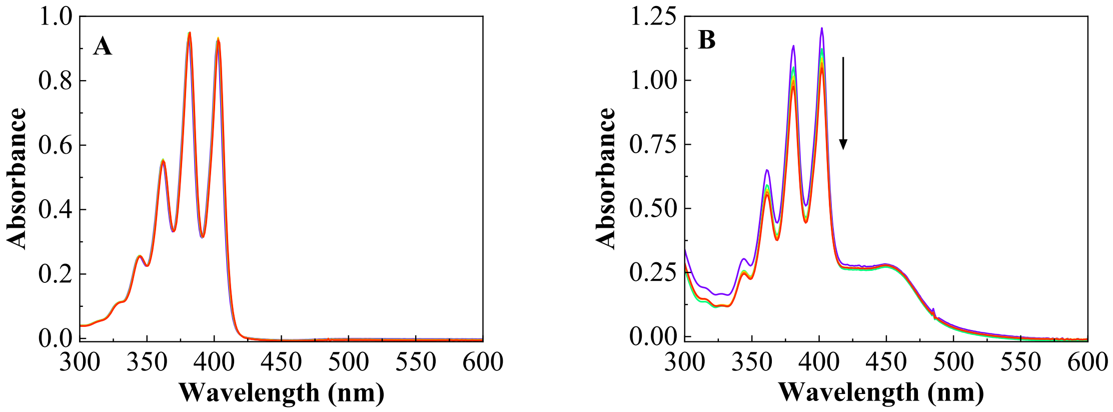

Figure S26: Bleaching of ABDA (100 μM) in DMSO upon irradiation at 450 nm in the presence ABDA control (A) and of the $[Ru(phen)_3]^{2+}$ reference (B) (irradiation time = 20 minutes).

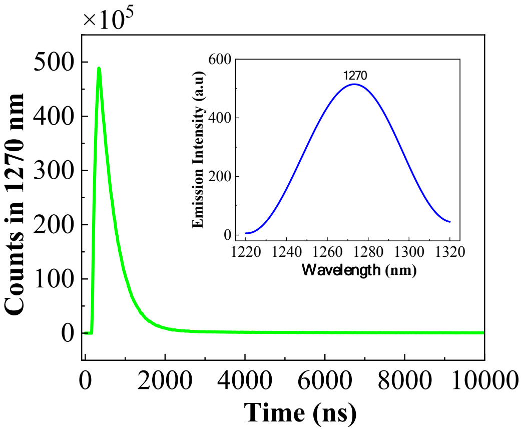

Figure S27. Emission transient at 1270 nm obtained after photoexcitation at 450 nm of a DMSO solution of $[Ru(phen)_3]^{2+}$.

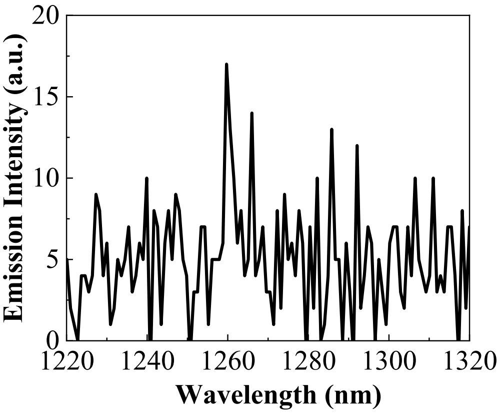

Figure S28. Emission transient at 1270 nm obtained after photoexcitation at 350 nm of a DMSO solution of pNDIp.

9. Photochemical Studies

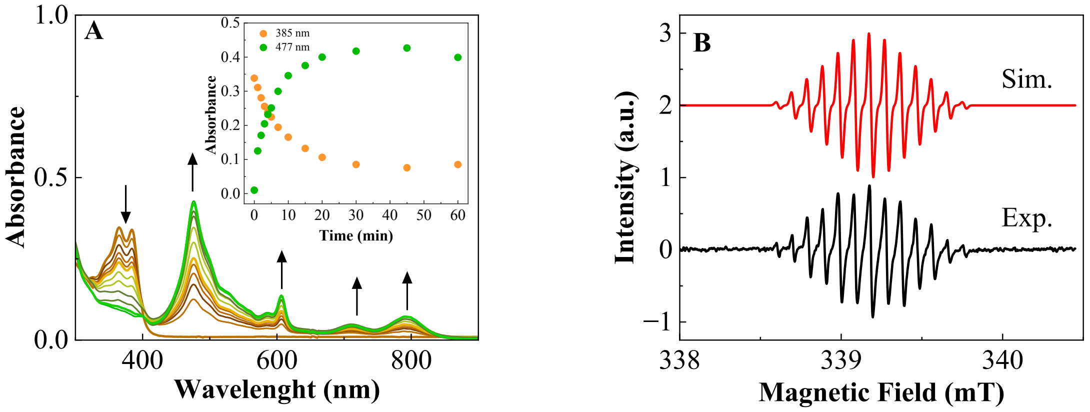

Figure S29. Photolysis of the pNDIp compound in deaerated DMSO solution with 350 nm irradiation light monitored by (A) absorption and (B) EPR spectrum with simulation.

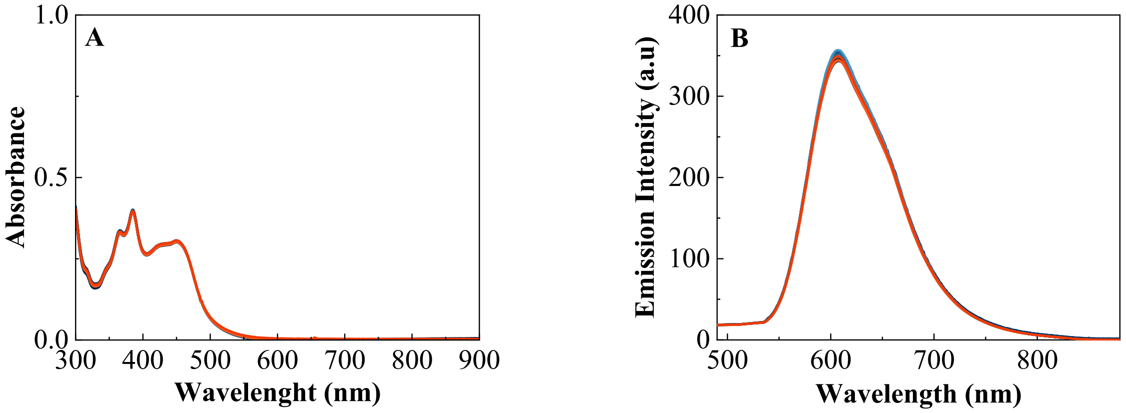

Figure S30. Photolysis of the RupNDIp dyad with 450 nm irradiation light in deaerated DMSO solution monitored by (A) Absorption and (B) Emission. (irradiation time = 180 minutes).

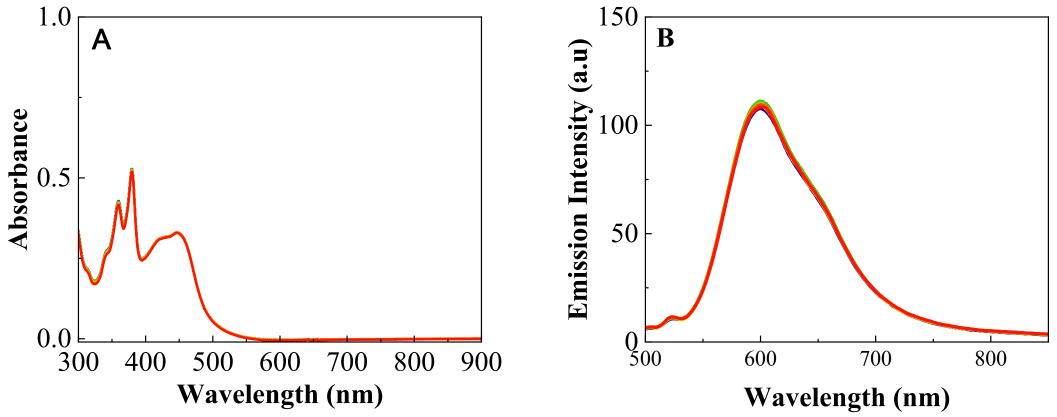

Figure S31. Photolysis of the RupNDIp dyad with 350 nm irradiation light in deaerated acetonitrile solution monitored by (A) Absorption and (B) Emission (λ_{EX}= 450 nm). (irradiation time = 180 minutes).

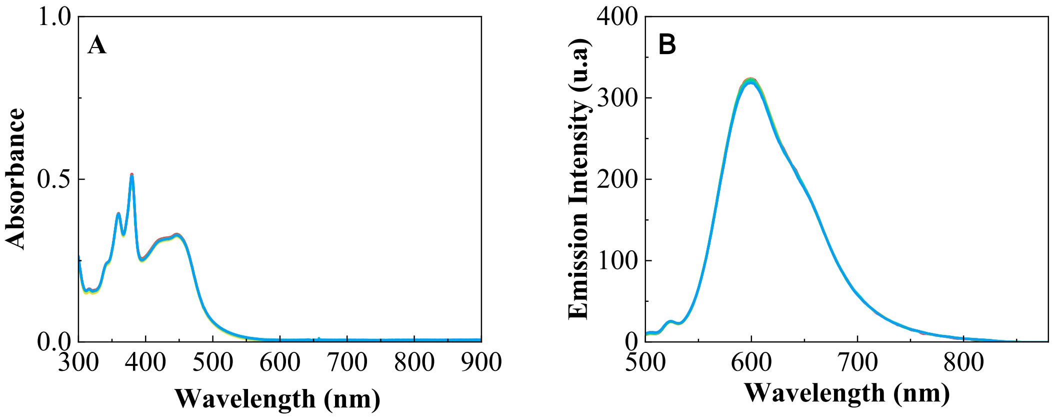

Figure S32. Photolysis of the RupNDIp dyad with 450 nm irradiation light in deaerated acetonitrile solution monitored by (A) Absorption and (B) Emission (λ_{EX}= 450 nm). (irradiation time = 180 minutes).

References

(1) Frisch, M. J.; Trucks, G.; Schlegel, H. B.; Scuseria, G. E.; Robb; Cheeseman, J. R.; Scalmani, G.; Barone, V.; Mennucci, B.; Petersson, G. A. Gaussian 09W, Revision A. 02; 2009.

(2) Lu, T.; Chen, F. Multiwfn: A Multifunctional Wavefunction Analyzer. *J Comput Chem* 2012, *33* (5), 580–592. https://doi.org/10.1002/jcc.22885.

(3) Lv, M.; Zhang, F.; Wu, Y.; Chen, M.; Yao, C.; Nan, J.; Shu, D.; Zeng, R.; Zeng, H.; Chou, S.-L. Heteroaromatic Organic Compound with Conjugated Multi-Carbonyl as Cathode Material for Rechargeable Lithium Batteries. *Sci Rep* 2016, *6* (1), 23515. https://doi.org/10.1038/srep23515.

(4) Marcus, R. A.; Sutin, N. Electron Transfers in Chemistry and Biology. *Biochimica et Biophysica Acta (BBA) - Reviews on Bioenergetics* 1985, *811* (3), 265–322. https://doi.org/10.1016/0304-4173(85)90014-X.

(5) Wu, S.; Zhong, F.; Zhao, J.; Guo, S.; Yang, W.; Fyles, T. Broadband Visible Light-Harvesting Naphthalenediimide (NDI) Triad: Study of the Intra-/Intermolecular Energy/Electron Transfer and the Triplet Excited State. *J Phys Chem A* 2015, *119* (20), 4787–4799. https://doi.org/10.1021/acs.jpca.5b01448.

(6) Hammarström, L. Artificial Photosynthesis: Closing Remarks. *Faraday Discuss* 2017, *198*, 549–560. https://doi.org/10.1039/C7FD00133A.

(7) Oevering, H.; Verhoeven, J. W.; Paddon-Row, M. N.; Cotsaris, E.; Hush, N. S. On a Long-Range Exchange Mechanism for Energy Transfer in Rigid Bichromophoric Molecules. *Chem Phys Lett* 1988, *150* (1–2), 179–180. https://doi.org/10.1016/0009-2614(88)80419-6.

(8) Oliver, A. M.; Paddon-Row, M. N.; Kroon, J.; Verhoeven, J. W. Orbital Symmetry Effects on Intramolecular Charge Recombination. *Chem Phys Lett* 1992, *191* (3–4), 371–377. https://doi.org/10.1016/0009-2614(92)85316-3.

(9) Creutz, C.; Newton, M. D.; Sutin, N. Metal—Lingad and Metal—Metal Coupling Elements. *J Photochem Photobiol A Chem* 1994, *82* (1–3), 47–59. https://doi.org/10.1016/1010-6030(94)02013-2.

(10) Jung, H. W.; Yoon, S. E.; Carroll, P. J.; Gau, M. R.; Therien, M. J.; Kang, Y. K. Distance Dependence of Electronic Coupling in Rigid, Cofacially Compressed, π-Stacked Organic Mixed-Valence Systems. *J Phys Chem B* 2020, *124* (6), 1033–1048. https://doi.org/10.1021/acs.jpcb.9b09578.

(11) Ratera, I.; Sporer, C.; Ruiz-Molina, D.; Ventosa, N.; Baggerman, J.; Brouwer, A. M.; Rovira, C.; Veciana, J. Solvent Tuning from Normal to Inverted Marcus Region of Intramolecular Electron Transfer in Ferrocene-Based Organic Radicals. *J Am Chem Soc* 2007, *129* (19), 6117–6129. https://doi.org/10.1021/ja066351g.
